# CDK1 Signaling in Cancer Treatment: Advances in AI-based Strategies and Tools for New Cancer Drug Discovery

**DOI:** 10.2174/0113862073426310251010113112

**Published:** 2026-01-12

**Authors:** Rajesh Basnet, Yujian Wu, Zhaoming Liu, Zhiyuan Li

**Affiliations:** 1 CAS Key Laboratory of Regenerative Biology, Guangdong Provincial Key Laboratory of Stem Cell and Regenerative Medicine, Guangzhou Institutes of Biomedicine and Health, Chinese Academy of Sciences, Guangzhou, 510530, China;; 2 University of Chinese Academy of Sciences, 19 Yuquan Road, Shijingshan District, Beijing 100049, China

**Keywords:** Cyclin-dependent kinases 1, artificial intelligence, cancer, cancer therapy, CDK1 inhibitors, crystal structure, drug design

## Abstract

Cyclin-Dependent Kinases (CDKs) are proteins that help control the cell cycle. They are considered potential targets for cancer treatment because they are often found at higher levels in cancer tissues than in normal tissues, and their presence is linked to survival rates in many cancer types. Cyclin-Dependent Kinase 1 (CDK1) is crucial for cell division and growth in cancer, as it significantly influences cell cycle progression through complexes formed with cyclins. Tumor growth can occur when CDK1 is deregulated, as its activation and phosphorylation of substrates are crucial for tumor development. Various small molecules that inhibit CDK1 have been developed and tested in preclinical studies, and some have progressed to human clinical trials. By inhibiting CDK1 activity, these drugs prevent it from changing other proteins and controlling the growth of cancer cells. Our study uses the STRING database, Kyoto Encyclopedia of Genes and Genomes (KEGG) pathway, and Gene Ontology (GO) analysis to reveal that CDK1 interacts with many proteins involved in cancer pathways. However, developing the best CDK1 inhibitors is challenging due to selectivity, potency, and cost, which are influenced by CDK1's structure and interactions with other proteins. This review explores the structure, function, regulation, mechanisms, and expression of CDK1, its crystal structure with various ligands, interactions with other proteins, and potential applications of CDK1 inhibitors. Future research, such as combination medicines, CRISPR, nanotechnology, and AI-driven methods and tools, should highlight their practical applications and provide a guide for efficient CDK1 discovery and drug development. Thus, this review emphasizes the significance of CDK1 targeting in cancer therapy, the difficulties in identifying potent inhibitors, and the ongoing research to enhance cancer treatment results by focusing on CDK1.

## 1 INTRODUCTION

The cell cycle is regulated by the oscillation of cyclins and Cyclin-Dependent Kinases (CDKs) [1]. Cyclin-Dependent Kinase 1 (CDK1) is one of about 20 CDKs and 30 cyclins in mammals. It is crucial for cell cycle control, especially in cancer. Removing CDK1 in mice leads to embryonic death, showing its vital and conserved role in cell cycle regulation across different organisms [2]. It manages the transition from the G2 phase to mitosis through interactions with cyclin B1 and phosphorylation processes [3, 4]. In the late G2 phase, the cyclin B1/CDK1 complex forms, ensuring the cell is prepared for mitosis after DNA replication checks [5]. When inactivated by PKR/EIF2AK2 and WEE1-mediated phosphorylation, CDK1 allows for DNA repair. CDK1 also phosphorylates NCKAP5L, leading to its dissociation from centrosomes during mitosis [4]. In humans, CDK1 activity is inhibited by Tyr15, which alters ATP orientation and hinders its kinase function [4]. Besides cell cycle regulation, CDK1 is involved in other essential biological processes, often working with cyclins A and E. Altered CDK1 expression is associated with cancer progression, and recent studies investigate the link between CDK1 phosphorylation and tumorigenesis [4, 6, 7].

Several new small-molecule Cyclin-dependent Kinase Inhibitor (CKIs) proteins have been developed in recent years. These include irreversible ATP-competitive drugs, structural inhibitors, CDK-degrading drugs, and inhibitory CDK-binding antibodies, highlighting the therapeutic potential of targeting CDKs in cancer treatment [8]. Ongoing research and clinical trials support targeting CDK1 as a promising therapeutic approach. In truth, it is clear that dysregulation and high levels of CDK1 are linked to aggressive tumors, making it an important target for cancer treatment [9]. Besides this, inhibiting CDK1 can stop cancer cell growth by arresting the cell cycle and inducing apoptosis, using specific inhibitors or combining with other anticancer drugs [7]. However, finding effective CDK1 inhibitors is still tricky because of variables like cost, potency, and selectivity influenced by CDK1's structure and interactions with other proteins.

This study explores the structure, domain organization, function, mechanisms, regulation, and activation of CDK1. Moreover, it also examines the role and expression profile of CDK1 in various cancers, CDK1 signaling pathways, and its protein interactions, providing insights from KEGG and GO term enrichment analysis. Furthermore, this review investigates ways to combat CDK1 inhibitor resistance, from the crystal structure to the group of selective inhibitors and their therapeutic significance. The study explores the development and current challenges associated with CDK1 inhibitors in cancer therapy, emphasizing their potential for clinical trials and FDA approval. However, resistance remains an issue, necessitating more research into combination therapy and emerging technologies such as CRISPR, nanotechnology, and AI-driven drug development to improve CDK1 inhibitors. Understanding resistance mechanisms and indicators is also critical for better cancer therapy with CDK1 inhibitors. Overall, this study emphasizes CDK1's crucial role in cancer and its potential as a therapeutic target while also addressing the hurdles and providing ways to improve treatment outcomes.

## 2 STRUCTURE AND DOMAIN ORGANIZATIONS OF CDK1

Structurally, CDK1 is a key player in the cell cycle, with a central part known as the catalytic core, which includes a cleft where ATP binds [10]. This binding allows CDK1 to transfer a phosphate group to its substrate, a pivotal step for the cell cycle to progress. Key amino acids involved in this process are Lys33, Asp145, and Glu91 [11, 12] (Fig. **1**). CDK1 contains a T-loop and a PSTAIRE helix. The T-loop blocks substrate binding when no cyclin is present. When a cyclin binds, these structures change, enabling CDK1 to perform its functions [4]. The crystal structure of CDK1 shows standard and unique features, including its ability to change shape when cyclin binds [13].

Moreover, many studies indicate that CDK1 must bind to a cyclin, usually Cyclin B, to become active. This binding occurs at the activation site, which includes the key residue Thr161. When a cyclin binds to CDK1, it induces a conformational change that exposes the catalytic site, allowing CDK1 to phosphorylate its substrates more effectively [4]. The polypeptide substrate binding site in CDK1 contains amino acids Arg50 and Arg51, which help anchor the substrate for accurate phosphorylation [14]. The ATP binding site, which is required for CDK1 activity, contains Glu81, Lys33, and Asp145 (Fig. **1**). These amino acids stabilize ATP, allowing it to donate a phosphate group to the substrate [10, 15].

On the other hand, human CDK1 shares about 63% of its amino acid sequence with its yeast counterpart and can even function with a specific mutation in yeast cells [16]. CDK1, like other kinases, has an aperture where ATP fits and a region where substrates bind near the aperture's opening. CDK1's structure ensures it can catalyze the addition of a phosphate group to its substrates effectively [10]. Understanding how these structural features allow CDK1 to function is critical for developing targeted cancer therapies. Focusing on these details allows researchers to develop treatments that specifically inhibit CDK1, potentially halting cancer cell growth and improving patient outcomes.

## 3 MECHANISMS, REGULATION, ACTIVATION, AND FUNCTION OF CDK1

The Serine/threonine protein kinase domain of CDK1 is tightly regulated through phosphorylation at specific amino acid residues, including Tyr-4, Ser-10, Ser-70, Thr-14, Tyr-15, and Thr-161 [4]. Regulatory proteins control CDK1 activity: activators such as PRKCD (Protein Kinase C Delta), PKMYT1 (Membrane Associated Tyrosine/Threonine 1), CDK2A (Cyclin-Dependent Kinase 2A), CDC25B (Cell Division Cycle 25B), PKR/EIF2AK2 (Protein Kinase R/Eukaryotic Translation Initiation Factor 2 Alpha Kinase 2), NPM (Nucleophosmin), NOL11 (Nucleolar Protein 11), CAK/CDK7 (CDK-Activating Kinase/Cyclin-Dependent Kinase 7), and LAR (Leukocyte Common Antigen-Related), and inhibitors like PTEN (Phosphatase and Tensin Homolog), MYT1 (Membrane Associated Tyrosine/Threonine 1), PTP1B (Protein Tyrosine Phosphatase 1B), CSN2B (Casein Beta), WEE1 (WEE1 G2 Checkpoint Kinase), WEE2 (WEE2 G2 Checkpoint Kinase), PRKCD (Protein Kinase C Delta), and CDKN1 (Cyclin-Dependent Kinase Inhibitor 1) [4]. This precise regulation of CDK1 through its kinase domain is essential for controlling cell division and presents potential targets for cancer therapies, as shown in Fig. (**2A**).

For the mechanism, CDK1 adds phosphate groups to other proteins, aiding in chromatin condensation, nuclear envelope breakdown, and spindle formation to ensure cells divide correctly. CDK1 is regulated by phosphorylation: a tyrosine (Tyr15 in humans) inhibits it by altering ATP orientation, stopping kinase activity. Kinases like Wee1 and PKMYT1 add inhibitory phosphates to CDK1 at Thr14 and Tyr15, and phosphatases like Cdc25A, Cdc25B, and Cdc25C remove these phosphates, fully activating the Maturation Promoting Factor (MPF) [4]. Cyclin B binds to CDK1, and CDK7 phosphorylates CDK1 at Thr161, activating the complex [17]. This active Cyclin B-CDK1 complex helps cells transition through the G2/M phase [18]. It is then inactivated by dephosphorylation and tagged Cyclin B for degradation by the APC/C (Anaphase-Promoting Complex/Cyclosome), ensuring proper cell cycle regulation and divisions [18], as shown in Fig. (**2B**).

Furthermore, CDK1 also affects microtubule dynamics by phosphorylating β-tubulins during mitosis and helps spindle formation through the phosphorylation of NEDD1 (Neural Precursor Cell Expressed [19], Developmentally Down-Regulated 1). The CDK1-cyclin B complex regulates processes like BMAL1 (Brain and Muscle ARNT-Like 1) expression and interaction with the HAUS (Augmin Complex) complex [20]. Surprisingly, cyclins control CDK1 by binding to it, changing access to its active site, and targeting it to specific cell locations. CKIs also regulate CDK1-cyclin complexes. Sic1 (S-phase Inhibitory Complex 1), a CKI, binds directly to Clb5,6-CDK1 (Cyclin B5,6-Cyclin-Dependent Kinase 1) complexes, timing S-phase entry by its destruction through ubiquitination. Its activity is regulated by phosphorylation at Thr161 by CDK-Activating Kinase (CAK), dephosphorylation at Thr14 and Tyr15 by CDC25 (Cell Division Cycle 25), and inhibition by CKIs like p16INK4a (Cyclin-Dependent Kinase Inhibitor 2A) [5].

Moreover, CDK1 interacts with various proteins, including cyclin B and Sox2, and is involved in cell division and cancer stem cell maintenance [21]. It adds phosphate groups to around 200 proteins, aiding gene expression, signal transmission, and cell division. In addition, it involves processes like signal transduction, apoptosis, mitochondrial function, Golgi organization, protein transport, and maintaining cellular balance [5]. During mitosis, CDK1 helps break down the nuclear envelope and condense chromatin, affecting gene expression. Furthermore, CDK1 promotes cell proliferation by modifying key signaling proteins like MAPK6 (Mitogen-Activated Protein Kinase 6) and ERK3 (Extracellular Signal-Regulated Kinase 3), while it regulates apoptosis by modifying proteins like caspase-9 and BIRC5 (Baculoviral IAP Repeat Containing 5). Additionally, it plays a role in mitochondrial dynamics, helps maintain cellular redox balance, and protects cells from oxidative stress [4]. Additionally, CDK1 modulates Golgi-related changes by phosphorylating proteins like GRASP65 (Golgi Reassembly Stacking Protein 65), GM130, and RAB1 (Ras-Related Protein Rab-1), causing Golgi disassembly. Similarly, it also regulates N-glycosylation enzymes, affecting glycosylation processes in the Golgi, and modifies proteins involved in endocytosis and nuclear transport [22]. Furthermore, by understanding these interactions and the CDK1-related pathways, researchers are developing targeted therapies that can inhibit CDK1, aiming to halt cancer progression and improve treatment outcomes.

## 4 EXPRESSION PROFILE OF CDK1 IN VARIOUS CANCERS

In over 20 human tumors, high CDK1 levels are linked to oncogenic genes, biological pathways, immune cell infiltration, tumor mutations, microsatellite instability, and lower survival rates, making CDK1 a promising target for cancer immunotherapy [23]. Moreover, its expression is often elevated in various cancers, including breast, colorectal, and liver [7]. However, in breast cancer, the expression of CDK1 varies among different breast cancer subtypes: Luminal A and B subtypes exhibit moderate CDK1 expression. In contrast, hormone receptors and Human Epidermal Growth Factor Receptor (HER2)-enriched subtypes show high CDK1 levels, leading to increased cell proliferation and poor outcomes [24]. Triple-Negative Breast Cancers (TNBCs) often have elevated CDK1 expression due to the absence of HER2 [25]. The Genotype-Tissue Expression (GTEx) project v8 shows that CDK1 is expressed in multiple human tissues, with high expression levels in the esophagus mucosa (16 TPM), skin (26 TPM), and testis (18 TPM). Other tissues, such as the liver, lungs, and various brain regions, show varying levels of CDK1 expression (Figs. **3A** , **B**).

The data from the Human Protein Atlas is presented in a bar graph, indicating the percentage of cancer patients with moderate to strong CDK1 levels in their cells. Most cancers, such as breast, colorectal, liver, lymphoma, melanoma, ovarian, pancreatic, skin, stomach, testis, thyroid, and urothelial cancers, exhibit high levels of CDK1. In contrast, prostate cancer shows positivity in a smaller fraction (Fig. **3C**). The Cancer Cell Line Encyclopedia's RNA-seq dataset of 1019 human cancer cell lines explains the CDK1 gene expression levels among diverse breast cancer cell lines. For example, HCC1428 has a high expression level at 286 TPM. In contrast, Hs 742.T has minimal expression, showing the unique significance of CDK1 in different breast cancer subtypes and its possible association with cancer development and treatment responses (Fig. **3B**). Apart from these, the impact of cytoplasmic CDK1 loss on gene expression and chemotherapy resistance in NSCLC is evident in the 'Meandiff plot' from the GSE28282 dataset, which compares gene expression changes between NSCLC cells and human bronchial epithelial cells. It reveals that losing cytoplasmic CDK1 is linked to poor survival and higher resistance to chemotherapy in lung cancer. The scatter of data points uniquely highlights which genes are most affected by CDK1 loss, suggesting potential targets for better lung cancer treatments. Moreover, this graph helps to understand how cytoplasmic CDK1 influences gene expression and treatment resistance in NSCLC (Fig. **3D**). Understanding how CDK1 functions and is expressed in different tissues and cell lines, as well as how the loss of cytoplasmic CDK1 affects gene expression, is critical for developing targeted cancer therapies. Using a raincloud plot for visual clarity, Fig. (**3E**) shows the distribution of CDK1 mRNA expression across different ER/PR (IHC) receptor statuses in cancer. The Differences in expression levels between ER+/PR+, ER+/PR-, ER-/PR+, and ER-/PR- groups are shown in box plots, and statistical analysis indicates that these differences are highly significant (**p < 0.0001**). These findings suggest CDK1's potential involvement in hormone-responsive cancer mechanisms and its impact on tumor progression.

## 5 CDK1 SIGNALING PATHWAYS AND ITS PROTEIN INTERACTIONS: INSIGHTS FROM KEGG AND GO TERM ENRICHMENT ANALYSIS

The CDK1 network and its associated proteins in cancer pathways, visualized using string databases, underscore the pivotal role of CDK1 in cell cycle control, particularly during G2-M and G1-S transitions. This research is of significant importance in the context of cancer studies. Briefly, CDK1 adds phosphate groups to proteins like actopaxin, APC, and CDC25, essential for cell cycle progression [26]. Meanwhile, WEE1 and PKMYT1 inhibit CDK1 by adding phosphate groups to prevent premature mitosis, while WEE2 inhibits CDK1 in oocytes to maintain meiotic arrest [27]. In addition, STIL affects CDK1 activity during development and growth. Furthermore, Cyclin-A2 (CCNA2) helps control cell cycle transitions by forming complexes with CDK1 or CDK2 (Cyclin-Dependent Kinase 2) [28]. Besides this, CDC25B (Cell Division Cycle 25B) and CDC25C (Cell Division Cycle 25C) activate CDK1 (Cyclin-Dependent Kinase 1) by removing inhibitory phosphates, and MNAT1 (Menage a Trois 1) helps form complexes that activate CDKs (Cyclin-Dependent Kinases) [26]. Similarly, Reprimo (RPRM) inhibits CDK1 to regulate cell cycle arrest [29], while Speedy A (SPDYA) activates CDK1 and CDK2 to help transition to the S phase and respond to DNA damage [30]. Likewise, CEP63 (Centrosomal Protein 63) is important for spindle assembly and centriole duplication, recruiting CDK1 to centrosomes. Furthermore, SPAG8 (Sperm Associated Antigen 8) delays CDK1 activation, assisting with spermatogenesis [31]. Many other proteins, such as ENSA (Alpha Endosulfine), maintain high CDK1 activity during mitosis by inhibiting PP2A (Protein Phosphatase 2A) [32]. LIN54 (Lin-54 Homolog), part of the DREAM complex (DP, RB-Like, E2F, and MuvB), regulates the CDK1 promoter [33]. RFPL1 (Ring Finger Protein Like 1) degrades cyclin B1 and CDK1 to manage the G2-M transition [26]. MASTL (Microtubule Associated Serine/Threonine Kinase-Like) maintains CDK1 activity during mitosis by inactivating PP2A [34]. In addition, Speedy C (SPDYC) helps activate CDK1 and CDK2 and plays a role in the spindle assembly checkpoint [35]. Moreover, RGCC (Regulator of Cell Cycle) enhances CDK1 activity and may help inhibit glioma growth by halt ing the cell cycle at G2/M [36].

 This study identified enriched biological terms using GO/KEGG pathways and other gene sets, then filtered them based on statistical significance and grouped them into clusters using Kappa-statistical similarities [37]. Each cluster is shown in an Excel sheet called "Enrichment Analysis." Key terms from these clusters were selected and visualized in a network layout, where nodes represent terms, sized by the number of related genes, and colored by cluster identity. Similar terms are connected by edges, with thickness indicating similarity. This comprehensive visualization highlights important biological processes. Protein-protein interactions were analyzed using a network, with GO enrichment analysis applied to identify key biological functions [38]. The MCODE algorithm helped find densely connected protein neighborhoods, extracting significant biological terms. This data shows the pathways and processes involved in the analyses [39].

Our enrichment analysis identifies critical signaling and regulatory pathways that recur with high negative scores, highlighting their biological significance. Both hormone-mediated and cell-cycle activities appear to be consistently enriched, implying that developmental and proliferative programs are under coordinated regulation. For example, "hsa04914" is associated with "Progesterone-mediated oocyte maturation" and has a value of -27.1, but "hsa04114" is associated with "Oocyte meiosis" and has a value of -25.8. The term "Cell cycle" (hsa04110) appears numerous times in different contexts, with varying enrichment values, indicating its significance. Similarly, "cell cycle phase transition" (GO: 0044770) and "mitotic cell cycle phase transition" (GO: 0044772) show notable enrichment, emphasizing their importance in the analyzed data (Fig. **4A**).

Fig. (**4B**) illustrates the interaction network of CDK1 and its associated proteins, highlighting the complex relationships and functional connections among them. Understanding the progesterone-mediated oocyte maturation pathway in depth, progesterone binds to GPCR2 (G Protein-Coupled Receptor 2) on the oocyte membrane, activating G proteins that stimulate AC (Adenylate Cyclase) to produce cAMP (Cyclic Adenosine Monophosphate). This cAMP activates PKA (Protein Kinase A), triggering the PI3K (Phosphoinositide 3-Kinase) and MAPK (Mitogen Activated Protein Kinase) pathways. PI3K leads to PIP3 (Phosphatidylinositol (3, 4, 5)-Trisphosphate) production and Akt/PKB (Protein Kinase B) activation. At the same time, the MAPK pathway involves proteins like Mos, MEK1 (Mitogen-Activated Protein Kinase Kinase 1), and pMAPK (phosphorylated Mitogen-Activated Protein Kinase), regulating key proteins such as CDK1 (previously called CDC2), Cyclin B (CycB), and APC/C. CDK1 and Cyclin B form the MPF essential for oocyte maturation. APC/C regulates the cell cycle by targeting proteins for degradation. Insulin and IGF-1 (Insulin-like Growth Factor 1) signaling also interact with this pathway, influencing oocyte maturation. The overall effect of this pathway is GVBD (Germinal Vesicle Breakdown) and progression through meiosis I and II, ensuring the oocyte matures properly for fertilization through crucial stages like G2 arrest, meiosis II entry, and metaphase II arrest. This research has practical implications for reproductive biology and potential applications in cancer therapy (Fig. **4C**).

Our study's KEGG analysis has revealed crucial pathways, including 'Progesterone-mediated oocyte maturation,' 'Cell cycle,' and 'Human T-cell leukaemia virus 1 infection.' These findings, depicted in Fig. (**5A**), not only indicate significant fold enrichment values but also underscore their profound significance in gene expression changes. Moreover, our protein expression data reveals tissue-specific profiles, with the pancreas pathway exhibiting the highest fold enrichment at 20-fold and an FDR of 2.2E-04 (Fig. **5B**). Similarly, our analysis of the human proteome expression, conducted using ShinyGO 0.77, has shown significant enrichment in pathways such as 'Fetal ovary' and 'Adult testis' (Fig. **5C**). Furthermore, our GO biological processes related to the cell cycle, like 'Regulation of mitotic sister chromatid separation' and 'Mitotic cell cycle phase transition,' are strongly represented (Fig. **5D**). The GO cellular component analysis highlights structures like the 'Cyclin A2-CDK2 complex' and 'Anaphase-promoting complex' (Fig. **5E**). Finally, the GO molecular function analysis reveals significant roles for 'Cyclin-dependent protein serine/threonine kinase activator activity' and 'Ubiquitin ligase activator activity' (Fig. **5F**). Fig. (**5G**) depicts the overall PPI network, providing a global view of the interactions among the proteins of interest. Fig. (**5H**) highlights the MCODE components, identifying densely connected modules within the network that may represent functional protein complexes. Fig. (**5I**) displays the enriched ontology clusters, colored by Cluster ID, to illustrate protein grouping based on shared biological functions. Fig. (**5J**) further illustrates the enriched ontology clusters, colored by p-value, emphasizing the statistical significance of these functional associations. Collectively, these analyses complement and are consistent with the KEGG and GO pathway enrichment results shown in Figs. (**4A**–**C**), thereby reinforcing the biological relevance of the identified protein interactions.

Our analysis of the string database data with FunRich has highlighted key characteristics of this study, each with significant practical implications. It displays protein domains where cyclin C and cyclin have the most considerable gene connections, emphasizing their importance in cellular activities. Other kinase-associated motifs like CKS, RHOD, STYKc, and S_TKc are present at more modest levels of 13.33% each, while smaller domains such as Mad3_BU and WD40 each account for less than seven percent (Fig. **5K**). Similarly, gene expression data indicate that the oral mucosa and serum exhibit considerable expression, suggesting these are important regions of interest. Rare occurrences in lymphoid stem cells register at only 5% apiece (Fig. **5L**). Furthermore, transcription factors such as NFYA (Nuclear Transcription Factor Y Subunit Alpha), KLF7 (Kruppel-like factor 7), and SP1 (Specificity Protein 1) are highly associated with a vast number of genes, implying regulatory involvement. Meanwhile, a smaller subset comprising CACD, NFE2, HOXA2, ZIC1, LHX8, ARNT, TEAD1, POUSF1, INSM1 and PAX5 each appears each account for 6.3% (Fig. **5M**). An inventory of somatic mutations in cancer shows that genes in the large intestine, lung, and biliary tract tumors are significantly affected by genetic changes. Lower mutation burdens are recorded in the biliary tract and cervix (Fig. **5N**). Finally, based on our analysis of different databases, we conclude that CDK1-integrating proteins are involved in signaling pathways in various cancers and the cell cycle. This understanding can inspire scientists to find new targets and create better cancer treatments, thereby advancing research and developing effective therapies. Furthermore, it clearly explains how proteins, genes, and mutations interact, simplifying cancer biology and research.

## 6 OVERVIEW OF CDK1, MEDIATORS, INHIBITORS, ONCOGENES, TUMOR SUPPRESSORS, AND CELL CYCLE SUBSTRATES

This comprehensive Table **1** highlights the key players in cellular regulation, including CDK1. It includes positive and negative mediators that influence a variety of biological processes, inhibitors now in preclinical and clinical trials, and critical elements involved in CDK1 activation and transcription. Table **1** also emphasizes important oncogenes, tumor suppressors, and cell cycle substrates, all of which play critical roles in cell cycle control and cancer development. Understanding these components allows us to gain insight into biological mechanisms, possible treatment targets, and disease development pathways. This comprehensive overview is an invaluable resource for academics and clinicians working in cellular biology and oncology.

## 7 ROLE OF CDK1 IN VARIOUS CANCERS

This review has so far focused on the pivotal role of CDK1 in various cancers. Our findings indicate that dysregulation of CDK1, whether due to mutations, overexpression, or changes in its regulatory partners, is associated with a range of cancers. These include uterine corpus endometrial carcinoma, colon adenocarcinoma, and skin cutaneous melanoma, with mutations found in about 0.74% of cancer patients [6]. CDK1's role in promoting cell growth and division is particularly pronounced in breast cancer, where high levels of CDK1 are linked to poorer outcomes [24]. Similarly, lung cancer exhibits more aggressive cells and a poorer prognosis when CDK1 is overexpressed [53]. In prostate cancer, CDK1 drives uncontrolled cell growth [9]. Acute Myeloid Leukemia (AML) also demonstrates that CDK1 aids cancer cells in multiplying [54]. However, pancreatic and colon cancers utilize CDK1 to develop tumors and resist treatment [55]. In the context of cancer, targeting CDK1 with specific inhibitors or combination therapies can enhance treatment outcomes by inhibiting uncontrolled cell growth and inducing cancer cell death, especially in tumors with high CDK1 expression [7, 9, 56]. These findings underscore the critical role of CDK1 signaling in cancer pathogenesis and the potential of CDK1 as a target for various cancer interventions.

### 7.1 Breast Cancer

In breast cancer, various growth factors activate CDK1, a protein that aids cell development. To address this, scientists developed CDK1 inhibitors. These medications are used to treat breast cancer that possesses hormone receptors but lacks the HER2 protein. In combination with hormone treatment, CDK1 inhibitors can enhance survival chances [57]. Nonetheless, some breast cancer cells may develop resistance to CDK1 inhibitors. This resistance occurs as CDK1 elevates the levels of specific proteins (members of the INK4 family) and forms a complex with another protein, p27^KIP1, hence diminishing drug sensitivity. CDK1 also influences the cell cycle by suppressing CDK2 [24]. Researchers employ CRISPR technology to delete CDK1 from breast cancer cells and study the resulting impacts. They discovered that CDK1 helps cancer cells copy and repair DNA, which increases the risk of cancer spreading [58]. Together, these points highlight the significance of CDK1 signaling in breast cancer pathogenesis.

### 7.2 Prostate Cancer

CDK1 can interact with the Androgen Receptor (AR), a protein that regulates masculine characteristics and prostate development. When CDK1 attaches to AR, it activates specific genes, and this interaction does not require CDK1's ability to modify other proteins [59]. However, cyclin D1 and p16INK4a proteins can inhibit CDK1 binding to AR. CDK1 also maintains a calm, inactive state in cancer stem cells, preventing them from being eliminated by the immune system or medicines. CDK1 interacts with NF-κB, a protein that regulates inflammation and cell survival, promoting cancer cell proliferation [60, 61]. Altogether, these points highlight the significance of CDK1 in prostate cancer.

### 7.3 Lung Cancer

CDK1 is a significant marker in lung cancer, namely Small-Cell Lung Carcinoma (SCLC). It predicts how the tumor will grow and how well the patient will respond to therapy [62]. The quantity of CDK1 can affect the progression of lung cancer. CDK1 levels are elevated in several malignancies, which may aggravate the condition. In SCLC, reduced CDK1 levels have been associated with improved results. CDK1 collaborates with CDK4 to control the cell cycle. They create a complex that determines whether a cell is ready to transition from the G1 to S phase [6]. If this complex is faulty or hyperactive, it can lead to cancer and aberrant cell proliferation. This shows that CDK1 is critical for the development of lung cancer, and that blocking CDK1 may be an effective therapy strategy [63].

### 7.4 Colon Cancer

Colon cancer cells frequently have an excess of cyclin D, which collaborates with CDK1 to initiate a pathway that promotes cancer development. CDK1 inhibits p27^KIP1, a protein that typically suppresses cancer cell division [64]. When CDK1 and cyclin D collaborate, they target p27^KIP1 for destruction, allowing cancer cells to divide. CDK1 also boosts the amounts of proteins such as Mcl-1 and Bcl-2, which assist cancer cells in surviving. CDK1 inhibitors, including palbociclib, ribociclib, and abemaciclib, can slow or halt the proliferation of colon cancer cells [55]. These medications work exceptionally well when combined with other therapies such as irinotecan or 5-fluorouracil. By attaching themselves to CDK1's active sites, CDK1 inhibitors prevent the protein from interacting with cyclin D, thereby stopping the cell cycle and killing cancer cells [7].

### 7.5 Pancreatic Cancer

CDK1 has an important role in 90% of pancreatic malignancies, particularly pancreatic ductal adenocarcinoma. It promotes cancer cell proliferation and survival by activating the KRAS protein, which regulates cell growth, differentiation, and survival. In 95% of PDAC patients, KRAS has a mutation that maintains it active at all times. CDK1 also inhibits a protein called p16INK4a, which ordinarily terminates the cell cycle. By identifying p16INK4a for destruction, CDK1 permits cancer cells to continue proliferating [65]. CDK1 renders DNA-damaging drugs such as gemcitabine and nab-paclitaxel less effective against cancer cells. CDK1 does, however, raise the amounts of proteins such as BRCA1 and BRCA2, which aid in the repair of cancer cells' DNA. CDK1 inhibitors, such as abemaciclib, can slow or halt the proliferation of pancreatic cancer cells, especially when paired with immunotherapy, PARP inhibitors, or ERK inhibitors [66]. CDK1 inhibition can benefit these therapies by decreasing the expression of KRAS, BRCA1, and BRCA2, and raising proteins that make cancer cells more apparent to the immune system [67].

## 8 CRYSTAL STRUCTURE DEVELOPMENT OF CDK1 WITH LIGANDS

The crystal structure of CDK1, a crucial enzyme for cell growth, has several key features. It includes a catalytic core for protein regulation, an ATP-binding pocket for energy, and N-terminal and C-terminal domains for interacting with other proteins (Fig. **1**). There is also a PSTAIRE-like domain for binding with cyclins [70]. Knowing and fully understanding this structure helps develop targeted therapies for diseases like cancer. Several crystal structures of CDK1 with different inhibitors reported in the literature, including key structures like the CDK1-Cks1 complex, reveal how CDK1 changes shape when binding with cyclins (Table **2**). This knowledge aids in designing effective inhibitors. In the literature, we found that some researchers have developed selective inhibitors targeting CDK1 by studying its interactions and binding properties. For example, the CDK1-cyclin B complex is less stable and more prone to dephosphorylation than CDK2-cyclin A, providing insights into how CDK1 functions. These findings are essential for creating cancer treatments targeting CDK1 [71]. Another cryogenic electron microscopy study has also shown how CDK1-cyclin B1 inhibits separase, ensuring proper chromosome segregation during cell division [72]. Mutagenesis studies highlight CDK1’s interactions with talin, linking cell proliferation and adhesion mechanisms [73]. Crystal structures of CDK1, especially in complex with cyclin B and regulatory subunits like CKS2, reveal several unique features that can guide the design of selective inhibitors [74] (Table **2**)**.** Compared to CDK2/Cyclin A, the CDK1/Cyclin B complex has a smaller interfacial surface and is less thermally stable, suggesting a more dynamic interaction that could be targeted by molecules disrupting this interface [75]. The active site of CDK1 also shows increased susceptibility to dephosphorylation at the activation segment, and subtle differences in substrate recognition motifs, which can be exploited to design inhibitors that preferentially bind CDK1 over other CDKs [76].

Additionally, the allosteric sites and conformational plasticity around the C-helix and activation loop offer opportunities for non-ATP competitive inhibition. These insights support fragment-based drug discovery and structure-guided design of compounds that either mimic native protein–protein interactions or selectively destabilize CDK1/Cyclin B assembly [77]. The potential of CDK1 as a target for cancer treatment is inspiring and should motivate further research in the field.

## 9 CURRENT STATUS OF LIGANDS FOR CDK1

Since the 1990s, CDKs have been crucial targets for cancer treatment, with clinical trials testing their inhibitors to improve outcomes for various cancers [44]. Given CDK1's role, several CDK1 inhibitors have been discovered and tested, as shown in Table **3**, and significant progress has been made in the treatment of cancer targeting CDK1. SGK1BEY1107, an orally active CDK1 inhibitor, has been assessed in phase 1/2 trials for its maximum tolerated dose, safety, and efficacy. It is proposed for use alone or with gemcitabine in advanced or metastatic pancreatic cancer [78]. Another class of drug called Flavopiridol, a pan-CDK inhibitor, has been used in clinical trials for treating leukaemia, multiple myeloma, sarcoma, gastrointestinal stromal tumors, and other solid tumors. Moreover, it is important to note that it has received an 'orphan drug' designation from the FDA (Food and Drug Administration) for LAML (Acute Myeloid Leukemia) [79]. Another drug, roniciclib, an orally bioavailable pan-CDK inhibitor, has shown promising results in trials for various neoplasms and lung cancer [80]. P276-00, a potent CDK inhibitor, has been evaluated for its potential in treating advanced refractory neoplasms, malignant melanoma, triple-negative breast cancer, and advanced head and neck cancer [81]. An additional drug to consider is dinaciclib, a selective CDK inhibitor that has shown significant effects in clinical studies, with positive responses in 54% of patients and a median progression-free survival of 481 days [80]. Similarly, AT7519a potent CDK1-associated inhibitor has shown promising anticancer activity against multiple cancer cell lines and tumor xenografts [82]. Overall, CDK1 inhibitors like BEY1107, Flavopiridol, Roniciclib, P276-00, Dinaciclib, AT7519, Seliciclib, AG-024322, PHA-793887, R547, RGB-286638, AZD-5438, and Indirubin, as shown in Table **3**, have shown potential for clinical application. However, safety and antitumor activity still need to be evaluated in future studies. In the literature found, the number of CDK1 inhibitors available is limited, and information about their selectivity is still unclear. Therefore, there is a high demand for further characterization of available tool compounds, exploring both selectivity and key protein-ligand interactions and the development of new CDK1 inhibitors.

## 10 COMPREHENSIVE STRATEGIES AGAINST CDK1 INHIBITOR RESISTANCE

Nowadays, CDK inhibitors have recently been developed and tested in clinical trials to enhance treatment and overcome resistance (Table **4**). They are frequently used in combination with therapy with hormones, chemotherapy, or targeted therapy [99]. The goal of ongoing research is to create more selective and effective CDK inhibitors as well as strategies to overcome resistance, including combination therapies, focusing on different pathways, identifying resistance biomarkers, improving the design of CDK1 inhibitors, continuous monitoring, adaptive treatment plans, and employing epigenetic modulators to increase CDK1 inhibitor sensitivity [100]. However, cyclin or CDK1 mutations, activation of other pathways, and overexpression of drug efflux pumps can all result in resistance (Table **4**). Cyclin E overexpression renders cancer cells resistant to CDK1 inhibitors by activating CDK2, enabling the cells to continue dividing even when CDK1 is blocked. Normally, CDK1 controls the cell's entry into mitosis, but when Cyclin E levels are high, CDK2 takes over part of CDK1's function, allowing the cell cycle to proceed properly [101]. This creates a backup system where CDK2 compensates for CDK1 loss. Furthermore, feedback loops involving proteins like Wee1 and Cdc25 regulate CDK1 activity, and Cyclin E/CDK2 can disrupt these loops, weakening the effect of CDK1 inhibitors [102]. Understanding these pathways is not just crucial; it is the key to developing strategies to overcome resistance and improve treatment outcomes [103]. Clinical trials focus on CDK1 to prevent cancer metastases and improve patient outcomes, while assessing the safety, effectiveness, dosage, and potential side effects of CDK inhibitors [104]. The emergence of acquired resistance to these inhibitors, frequently linked to modifications in cell cycle regulatory pathways, poses a severe problem despite their initial effectiveness. Because of this resistance, patients may become indifferent to treatment, leading to the need for an improved understanding of the underlying mechanisms [105]. It can determine which tumors are most responsive to treatment and develop possible combinatorial approaches by examining these pathways and their contributions to resistance.

Inhibitors targeting alternative compensatory signaling pathways may be one of these tactics; this would help overcome drug resistance and improve the therapeutic efficacy of CDK1 inhibitors [106]. To summarize, research and clinical trials are critical for completely understanding resistance mechanisms and developing novel medicines, which will considerably improve the efficacy of CDK1 inhibitors in cancer treatment.

## 11 FUTURE OF CDK1 INHIBITORS IN CANCER THERAPY

Flavopiridol and Seliciclib were among the first drugs made to block CDKs, but they were found to be problematic because they did not target just CDK1; they hit multiple CDKs like CDK2, CDK4, and CDK9 (Tables **3** and **5**) [107-111]. This led to serious side effects such as diarrhea, liver damage, and low white blood cell counts, making them dangerous at doses high enough to work [79, 112]. These drugs also did not stay long in the body and were poorly absorbed, which made treatment more complicated. It is hard to make a drug that blocks only CDK1 because its active site looks very similar to those of other CDKs, especially CDK2, so many drugs end up hitting multiple targets. CDK1 also changes shape depending on which cyclin it is attached to, which makes drug design even more difficult [113]. All of these factors explain why it is tough to create a safe and effective CDK1-specific drug for cancer. Other CDK inhibitors, such as Roscovitine, Dinaciclib, AT7519, Palbociclib, and Ribociclib, also faced similar issues. Early inhibitors such as Flavopiridol and Roscovitine blocked multiple CDKs and caused dose-limiting problems like liver damage, immune cell loss, and heart side effects. Dinaciclib and AT7519 were better designed but still had trouble with selectivity and tolerability [113, 114]. Palbociclib and Ribociclib are used for breast cancer, but they mainly target CDK4 and CDK6, not CDK1, and only work for specific cancer types (Table **3** and Table **5**) [115]. The biggest challenge is that the ATP-binding pocket is very similar across CDK family members, especially CDK1 and CDK2, making it difficult to design a drug that targets only CDK1. Additionally, CDK1 changes shape when it binds to different cyclins, which makes the drug-binding surface harder to predict [113]. Because of all these problems, researchers are now exploring new ways, such as allosteric inhibition and targeting protein interactions, to develop more selective CDK1 drugs.

Table **5** provides a comprehensive overview of advanced clinical studies on first, second, and third-generation CDK1 inhibitors in cancer therapy, highlighting the advancements and research focus on distinct CDK1 inhibitors, including their chemical structures, targeted CDKs, and clinical trial stages. CDK1 inhibitors are being developed to boost therapy efficacy by targeting CDK1 while ignoring other CDKs or kinases [9, 44, 49]. Combination treatment is being studied to improve the effectiveness of CDK1 inhibitors and overcome resistance mechanisms [116]. Nanotechnology is being used to deliver CDK1 inhibitors to tumor cells more efficiently, improving pharmaceutical solubility, stability, and targeted delivery [117]. Biomarker identification is used to predict medication response and identify patients who are more likely to benefit [118]. CRISPR/Cas9 technology is being used to examine CDK1's function in cancer and identify potential resistance mechanisms [119, 120]. CDK1 inhibitors are being designed and optimized using artificial intelligence and machine learning, with vast datasets analyzed to discover prospective therapeutic candidates and forecast efficacy and safety [58, 121]. In HR+ or HER2-positive malignancies, which are prevalent in breast, prostate, and endometrial cancers, patients frequently develop resistance to Endocrine Treatment (ET), resulting in relapses and lifelong chemotherapy. To improve ET efficacy, researchers are developing drugs targeting critical cellular growth and regulatory pathways, with CDKIs among these novel therapies, which assist in regulating the cell cycle and limiting cancer cell multiplication [122]. CDK1 and other CDK inhibitors have demonstrated potential in treating advanced tumors. Ongoing research aims to further understand and optimize their efficacy in contemporary cancer therapy, providing new hope for patients with HR+ cancers.

## 12 ADVANCES IN AI-BASED STRATEGIES AND TOOLS TO FACILITATE CDK1 IN FUTURE DRUG DEVELOPMENT

Artificial Intelligence (AI) is revolutionizing drug development by targeting CDK1 [123]. AI-driven approaches enhance drug discovery and development stages, allowing researchers to prioritize CDK1 as a therapeutic target [124]. By integrating data from diverse sources, AI accelerates the identification and understanding of CDK1's mechanisms, enabling more effective treatments. This advancement is accelerating the development of CDK1 inhibitors, offering promising cancer treatment avenues [125] (Fig. **6**). AI technologies are increasingly used in drug discovery to predict protein structures, simulate molecular interactions, and identify potential drug candidates. Tools like AlphaFold2 help model the 3D shape of proteins such as CDK1, which is crucial for accurate docking studies [126]. Deep learning frameworks like DeepChem [127] and Chemprop are used to screen large molecular libraries and predict how well compounds might inhibit target proteins [128]. These models use datasets from ChEMBL or BindingDB to estimate parameters like IC_50_ or binding affinities. This helps researchers pick drugs for further testing. Using well-established platforms and focusing on specific cancer targets can improve the effectiveness and scientific validity of AI integration.

### 12.1 Target Identification

AI is utilizing multiomics data to identify potential drug targets [129, 130]. This data, integrating genomics, transcriptomics, proteomics, and metabolomics, helps understand how CDK1 dysregulation contributes to oncogenic processes like uncontrolled cell proliferation and tumor development [130-132]. Machine learning algorithms help identify patterns and correlations within the data, making it easier for AI to detect them than for humans [133, 134].

- **Genomic Analysis:** AI investigates mutations or changes in genes associated with CDK1 activity, highlighting its role in specific cancer subtypes [135].
- **Transcriptomic Insights:** Advanced tools measure changes in CDK1 expression levels across tissues, determining its role in tumor progression [136-138].
- **Proteomic Mapping:** AI identifies post-translational modifications and protein-protein interactions involving CDK1, which are critical for developing targeted therapies [139, 140].
- **Metabolomic Profiling:** It assesses the metabolic pathways influenced by CDK1, linking its activity to broader cellular dysfunctions in cancer [7].

### 12.2 Structure-Based Drug Design

AlphaFold, an AI-powered tool, is a critical tool in the field of Structure-Based Drug Design (SBDD), which employs detailed knowledge of a target protein's three-dimensional structure to create molecules that modulate its function [141, 142]. This method is beneficial for developing selective inhibitors of CDK1, a protein that regulates the cell cycle. SBDD has great potential for cancer therapy by accelerating the discovery of CDK1 inhibitors, improving their precision and effectiveness, and targeting CDK1's dysregulated activity [9, 99].

- **High-Resolution Protein Modeling:** AlphaFold creates precise 3D models of CDK1, capturing key structural details like the active site, binding pockets, and conformational states. This knowledge is essential for understanding how small molecules interact with CDK1 [143, 144].
- **Ligand Docking Studies:** With the structural model in hand, computational methods are used to simulate the binding of potential drug candidates (ligands) to CDK1. These simulations aid in determining the most promising compounds based on their binding affinity, stability, and specific interactions, such as hydrogen bonding and hydrophobic effects [7, 145].
- **Selective Binding Design:** Given the structural similarities between CDK1 and other cyclin-dependent kinases, achieving selectivity presents a significant challenge. AI tools can analyze subtle differences in binding sites, allowing molecules to be designed that specifically target CDK1 while minimizing off-target effects [146].
- **Optimization of Lead Compounds:** Once initial candidates have been identified, structure-based optimization refines their chemical structures to improve potency, selectivity, and pharmacokinetics. AI speeds up the iterative process by predicting changes that will enhance drug-likeness [130, 147].
- **Dynamic Simulations:** Molecular dynamics simulations reveal insights into CDK1's flexibility and movements, ensuring that inhibitors bind effectively under physiological conditions. These simulations are critical for predicting possible resistance mechanisms [148].

### 12.3 Virtual Screening

Virtual screening, powered by AI, is a game-changing approach to drug development, allowing researchers to efficiently analyze large chemical libraries to identify potential inhibitors for targets such as CDK1. This procedure not only saves time but also lowers the costs associated with conventional experimental screening methods. AI-driven virtual screening accelerates the identification of potential CDK1 inhibitors while also improving precision and specificity [149]. This approach is proving invaluable in the development of targeted therapies for cancers in which CDK1 dysregulation is a major factor.

- **Chemical Space Exploration:** AI algorithms can search vast libraries containing millions or even billions of compounds. These libraries contain both synthetically known molecules and hypothetical structures created computationally. By sifting through such a large chemical space, AI can identify compounds that are highly likely to bind to CDK1's active site [149, 150].
- **Predictive Modeling:** Machine learning models trained on experimental data use molecular structure, functional groups, and electronic properties to predict compound binding affinity to CDK1 [151-153]. These models are frequently refined through reinforcement learning to improve accuracy over time.
- **Similarity-Based Screening:** AI uses techniques like molecular fingerprinting and graph-based representations to identify compounds that are structurally similar to known CDK1 inhibitors [146, 154]. This allows researchers to identify analogs with higher efficacy or fewer side effects.
- **Binding Site Assessment:** AI-powered docking programs simulate compound interactions with CDK1's binding pockets. These simulations take into account hydrogen bonds, van der Waals forces, and electrostatic interactions when estimating binding strength and stability [149, 155, 156].
- **Scoring and Ranking:** Once binding interactions have been modeled, AI assigns a score to each compound based on its predicted binding affinity and pharmacokinetic properties. Compounds with the highest scores are prioritized for experimental validation, which greatly simplifies the selection process [157-159].
- **Integration of Multiomics Data:** AI improves virtual screening by incorporating insights from multiomics analyses (genomics, proteomics, *etc*.) to ensure that shortlisted compounds target biologically relevant CDK1 pathways [159, 160].
- **Lead Compound Optimization:** After identifying promising candidates, AI helps to optimize their structures to improve drug-like properties like solubility, permeability, and metabolic stability, while maintaining strong interactions with CDK1 [161, 162].

### 12.4 De Novo Drug Design

Machine learning algorithms generate optimized molecular structures. De novo drug design is a cutting-edge approach in medicinal chemistry that involves creating novel molecular structures from scratch to target specific proteins, such as CDK1. Using machine learning algorithms, this method combines computational creativity and biological precision to create optimized compounds with therapeutic potential. This novel approach to drug design is changing how researchers develop targeted therapies for diseases such as cancer [163, 164]. Scientists can use AI to explore an almost infinite chemical space, discovering molecules with unprecedented potential to inhibit CDK1 and contribute to better treatment outcomes.

- **Defining the Target:** De novo drug design begins with obtaining detailed information about CDK1, such as its 3D structure, active sites, binding pockets, and molecular interactions. AI tools such as AlphaFold provide structural data required for designing molecules that perfectly align with CDK1's functional regions [165, 166].
- **Generating Novel Molecular Scaffolds:** AI algorithms use techniques like Generative Adversarial Networks (GANs) and reinforcement learning to propose completely new chemical structures. These scaffolds are designed to fit CDK1's binding site while avoiding structural similarities with existing compounds, increasing the likelihood of finding novel inhibitors [167, 168].
- **Predicting Binding Affinity:** Once the initial set of molecules has been generated, AI models predict how well they will interact with CDK1. Molecular docking simulations calculate binding strength, specificity, and stability to help prioritize the most promising candidates [156, 169, 170].
- **Optimizing Pharmacokinetic Properties:** AI refines lead compounds to improve drug-like properties such as solubility, bioavailability, and metabolic stability. These improvements ensure that the molecules not only inhibit CDK1 but also work well *in vivo* [4, 171].
- **Balancing Efficacy and Selectivity:** Given CDK1's similarities to other cyclin-dependent kinases, achieving selectivity is a significant challenge. Machine learning algorithms evaluate molecular designs based on their ability to specifically target CDK1 while minimizing off-target effects, thereby reducing potential side effects [4].
- **Iterative Design Cycles:** AI accelerates the traditional iterative process of molecular tweaking by continually learning from simulation results and experimental feedback. This accelerates the development of compounds that meet the preclinical standards [168].
- **Integration with Biological Data:** De novo design incorporates biological insights, such as multiomics data, to ensure that the proposed molecules not only bind to CDK1 but also affect cancer-related pathways [4, 7].

### 12.5 Clinical Trial Optimization

AI enhances trial design through predictive modeling and adaptive strategies, resulting in more efficient testing of targeted therapies. Clinical trial optimization is critical for speeding up the development of CDK1-targeted therapies and ensuring that promising treatments reach patients quickly and safely. AI improves trial design, execution, and analysis through predictive modeling and adaptive strategies [172, 173]. Researchers can more effectively test CDK1-targeted therapies by incorporating AI into all stages of clinical trial design and execution, paving the way for innovative cancer treatments.

- **Predictive Modeling for Patient Recruitment:** Finding the appropriate patients is one of the most challenging aspects of clinical trials. AI analyzes a variety of datasets, including electronic health records, genetic profiles, and biomarker data, to identify people who could benefit from CDK1 inhibitors. This ensures that trials are focused on populations with the highest likelihood of success, thereby improving efficacy and lowering costs [172, 174].
- **Adaptive Trial Design:** AI-powered adaptive designs, known for their real-time adjustments based on interim results, are a game-changer in clinical trials. For instance, if a specific CDK1 inhibitor demonstrates strong efficacy in certain patient subgroups, the trial protocol can be swiftly modified to concentrate on those subgroups. Similarly, if adverse effects are identified early, AI can promptly guide dosage adjustments and refine inclusion criteria, thereby reducing risks [173, 175].
- **Optimal Dosing Strategies:** Researchers can use AI simulations to predict the optimal dosing regimens for CDK1 inhibitors, balancing therapeutic efficacy and safety [4, 7, 176]. This shortens the time and resources required for dose-finding studies.
- **Real-Time Monitoring and Data Analysis:** During trials, AI systems examine incoming data to identify patterns and anomalies. This enables researchers to recognize early signs of efficacy or resistance [4, 177]. Address safety concerns proactively to reduce the likelihood of trial failure due to unforeseen issues.
- **Biomarker Identification:** AI aids in the discovery of predictive biomarkers related to CDK1 activity, allowing trials to be tailored for patients with specific genetic or molecular profiles. This precision medicine approach improves the efficacy of targeted therapies [7, 178].
- **Streamlining Regulatory Compliance:** AI assists in managing the massive documentation required for regulatory approval, ensuring that trial protocols, patient data, and results meet rigorous standards [179-181].
- **Cost and Time Efficiency:** AI reduces the cost and time of clinical trials by automating data analysis and enabling adaptive strategies. This acceleration is beneficial for developing CDK1 inhibitors, where timely intervention can improve patient outcomes [4, 102, 182].
- **Patient Retention and Engagement:** AI tools, such as chatbots or apps, can help participants communicate more effectively, keeping them informed and engaged throughout the trial. This reduces dropout rates and ensures the study's integrity [4, 183].

### 13 CHALLENGES WITH CDK1 INHIBITORS IN THE PAST, PRESENT, AND FUTURE

Over the last few decades, three generations of CDK1 inhibitors have been created. The first and second generations exhibited strong anticancer efficacy in lab studies; however, they lacked high selectivity, targeting multiple CDKs, which led to increased toxicity and impaired normal cell functioning [184]. This resulted in considerable side effects, making them less appealing for cancer therapy. On the other hand, the third-generation CDKIs, notably CDK1 inhibitors, have shown more promise, with clinical studies and FDA approval [185]. However, individuals continue to confront hurdles such as innate and learned resistance. Some patients may not react to these advanced CDKIs, while others develop resistance after early success. Studies have discovered different resistance mechanisms, including genetic alterations, changes in essential cell cycle regulators, and activation of survival pathways [186]. Despite several laboratory investigations, we still require additional clinical data on these resistance pathways. To address this, further research is needed to better understand resistance, develop biomarkers to identify individuals who would benefit from these medications, and apply combination methods with other treatments to increase the effectiveness of CDK1 inhibitors in cancer treatment. CDK inhibitors that target cancer cells are critical for various cancer treatments, especially in MYC-dependent cancers, where MYC plays an important role in oncogenic processes such as proliferation, apoptosis, differentiation, and metabolism. To achieve a comprehensive and sustainable solution, understanding CDK regulation is essential for developing effective inhibitors [49]. CDK1 inhibitors can reduce tumor growth and proliferation; however, their potential to replace traditional endocrine therapies is limited by side effects, toxicity, resistance, and lower effectiveness [187]. These findings highlight the importance of understanding how CDKs function in cancer and normal cells to balance their benefits and risks. Since CDK1 inhibition has proven successful in treating oesophageal, lung, and prostate cancer, as well as relapsed or refractory chronic lymphocytic leukaemia, combination treatments with other anticancer agents may help address these challenges [188]. Developing inhibitors specifically targeting cancer cells while causing limited damage to normal cells necessitates a complete understanding of CDK control. However, CDK1 inhibitors have not always proven useful in cancer patients, likely due to poor pharmacokinetics or low CDK1 expression levels. Besides, screening for high CDK1 expression is crucial for patient recruitment, and combination therapy may improve the efficacy of CDK1-associated inhibitors in clinical trials.

## 14 FUTURE DIRECTIONS AND EMERGING TRENDS

Nowadays, advancements in AI are rapidly shaping kinase drug discovery, with a growing interest in AI-guided selectivity profiling. This approach is designed to find and improve target precision and minimize off-target effects [189, 190]. Several machine learning frameworks, including MolMapNet, Chemprop, and DeepKinZer, are being applied to predict kinase-inhibitor interactions across large compound libraries. These models, which utilise protein sequence embeddings, chemical fingerprints, and structural data, are capable of ranking compounds based on selectivity scores, even for understudied kinases [191]. Despite the difficulties associated with CDK1 selectivity prediction, notably because of its conserved ATP-binding site, new deep learning techniques are being developed that utilize tiny interfacial and conformational differences for customized design [146]. AI is playing a crucial role in overcoming these challenges and is a reassuring sign of progress in the field. A deeper understanding of compensatory mechanisms and resistance evolution is made possible by the integration of explainable AI techniques with multi-omics datasets, such as phosphoproteomics and CRISPR screens [192]. Through better treatment windows and more efficient candidate selection, these technologies have the potential to speed up precision oncology.

## CONCLUSION

In this review, we discuss the function and mechanisms of CDK1 in cancer. Targeting CDK1 can provide opportunities for cancer prevention and treatment. Combining CDK1 inhibitors with other anticancer drugs may enhance chemotherapy benefits and improve clinical outcomes for cancer development. CDK1, which is highly expressed in various tissues and cell types, influences key cancer processes such as cell proliferation, death, invasion, and spread. We also studied proteins that interact with and/or are mediated by CDK1. Our findings showed that CDK1-associated proteins are essential in a variety of cancer signaling pathways. Despite its importance in cancer, CDK1’s mitotic function is well established, but its non-canonical roles in cancer, such as gene regulation, stress response, and apoptosis, remain unclear. Promising future technologies, such as CRISPR gene editing, nanotechnology, and AI-driven drug discovery, may further refine CDK1 inhibitor treatments. This could lead to more effective and tailored medicines, improved patient outcomes, and an extended therapeutic potential for CDK1 inhibitors. The study of existing CDK inhibitors is a crucial part of this research, aiding in understanding cancer pathways and developing new treatments.

## Figures and Tables

**Fig. (1):**
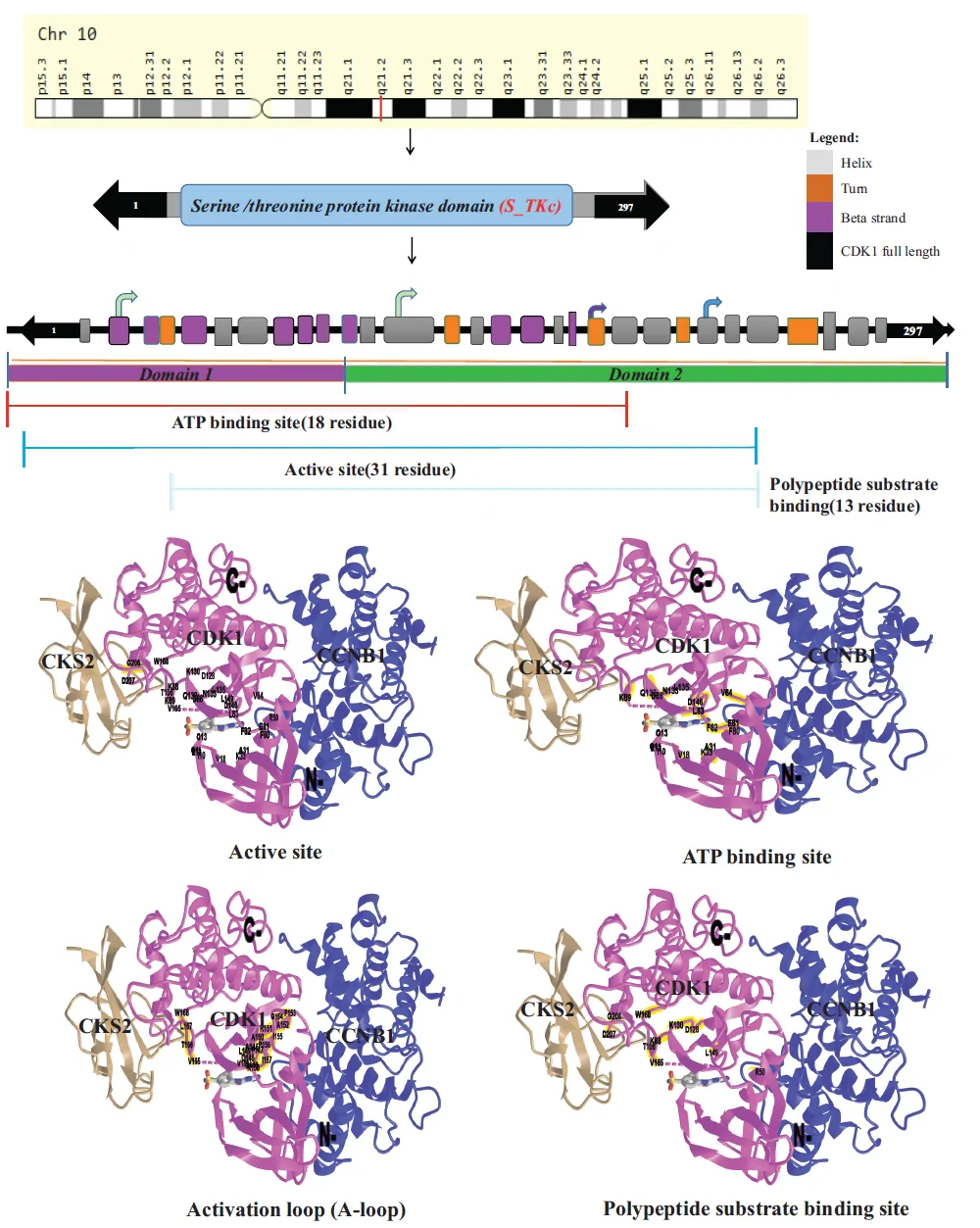
Structural and domain organization of monomeric CDK1 kinase domain with functional site (PDB ID: 5LQF).

**Fig. (2):**
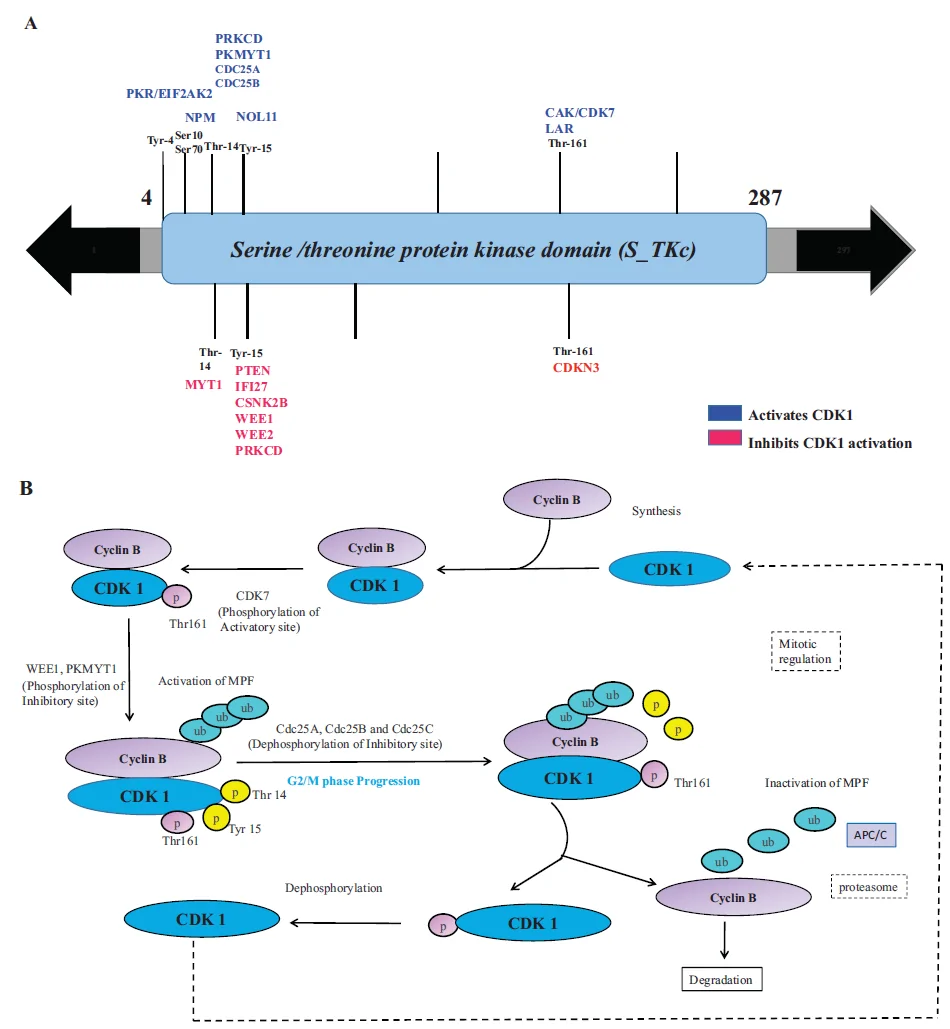
Mechanism and regulation sites of CDK1 activation. (A) Regulatory Sites of CDK1: Illustrates key phosphorylation sites and regulatory partners, including inhibitory and activating kinases. (B) Mechanism of CDK1 Activation.

**Fig. (3):**
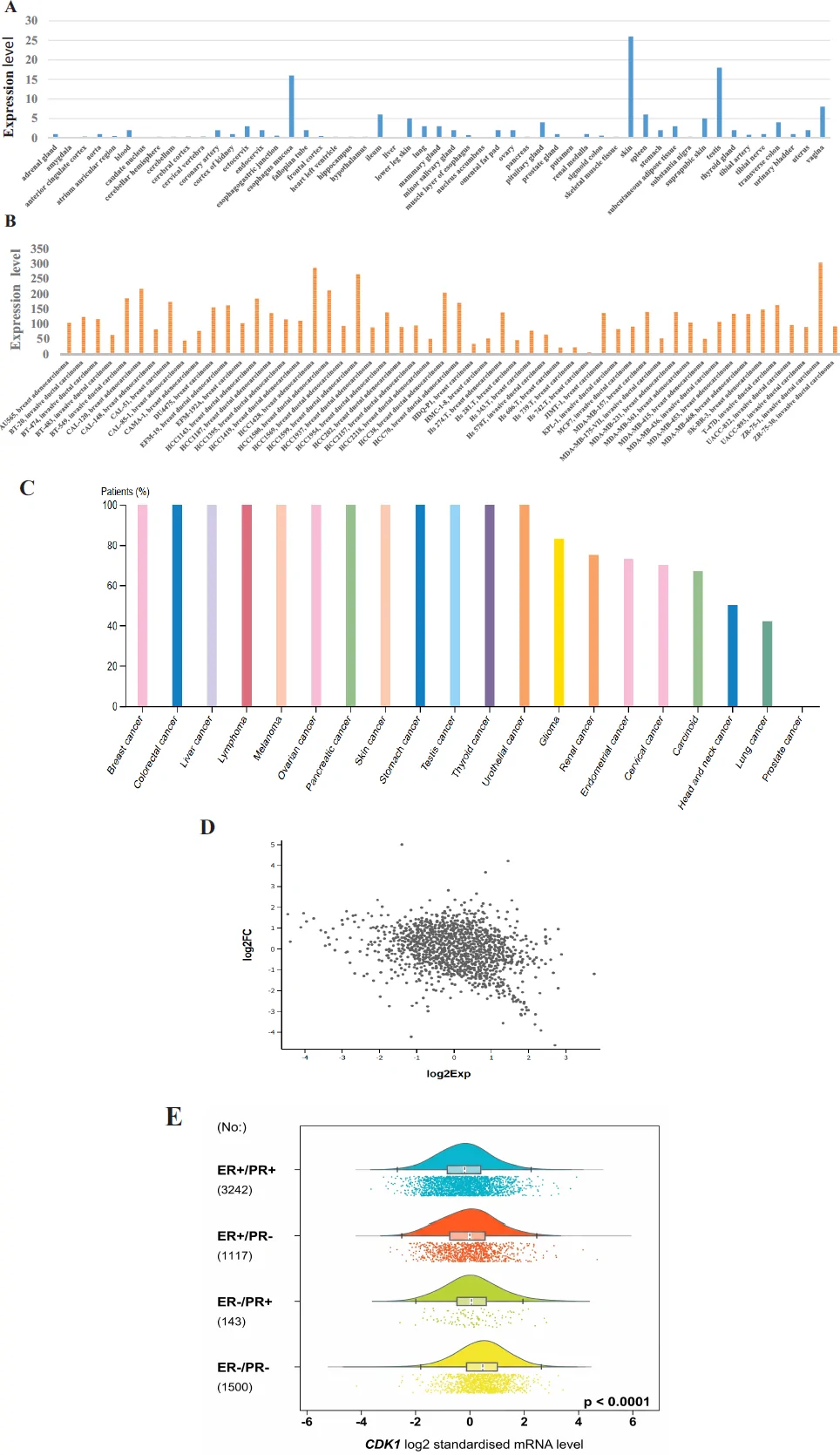
Expression profile of CDK1. (A) Data from the Genotype-Tissue Expression (GTEx) project v8 showing CDK1 levels in various human tissues. (B) RNA-seq data from the cancer cell line encyclopedia explaining CDK1 expression in different breast cancer cell lines. (C) Bar graph from the Human Protein Atlas indicating the percentage of cancer patients with moderate to strong CDK1 levels. (D) A meandiff plot shows the difference in average values between two groups. 'Meandiff plot' from the GSE28282 dataset showing the relationship between log2 fold change (log2FC) and log2 expression (log2Exp) in Non-Small Cell Lung Cancer (NSCLC) cells *versus* human bronchial epithelial cells, highlighting significant changes in gene expression and potential therapy targets. (E) A raincloud plot shows data distribution using a combination of raw points, boxplot, and density curve, all in one compact visual. Raincloud plot depicting CDK1 mRNA expression across different ER/PR (IHC) statuses in cancer. The distribution of log2 standardized CDK1 levels is visualized for four groups: ER+/PR+ (3262 samples), ER+/PR- (283 samples), ER-/PR+ (45 samples), and ER-/PR- (462 samples). Box plots highlight the median and interquartile ranges, with statistical analysis indicating significant differences (p < 0.0001) in CDK1 expression between receptor subtypes. The data is available in bc-GenExMiner.

**Fig. (4):**
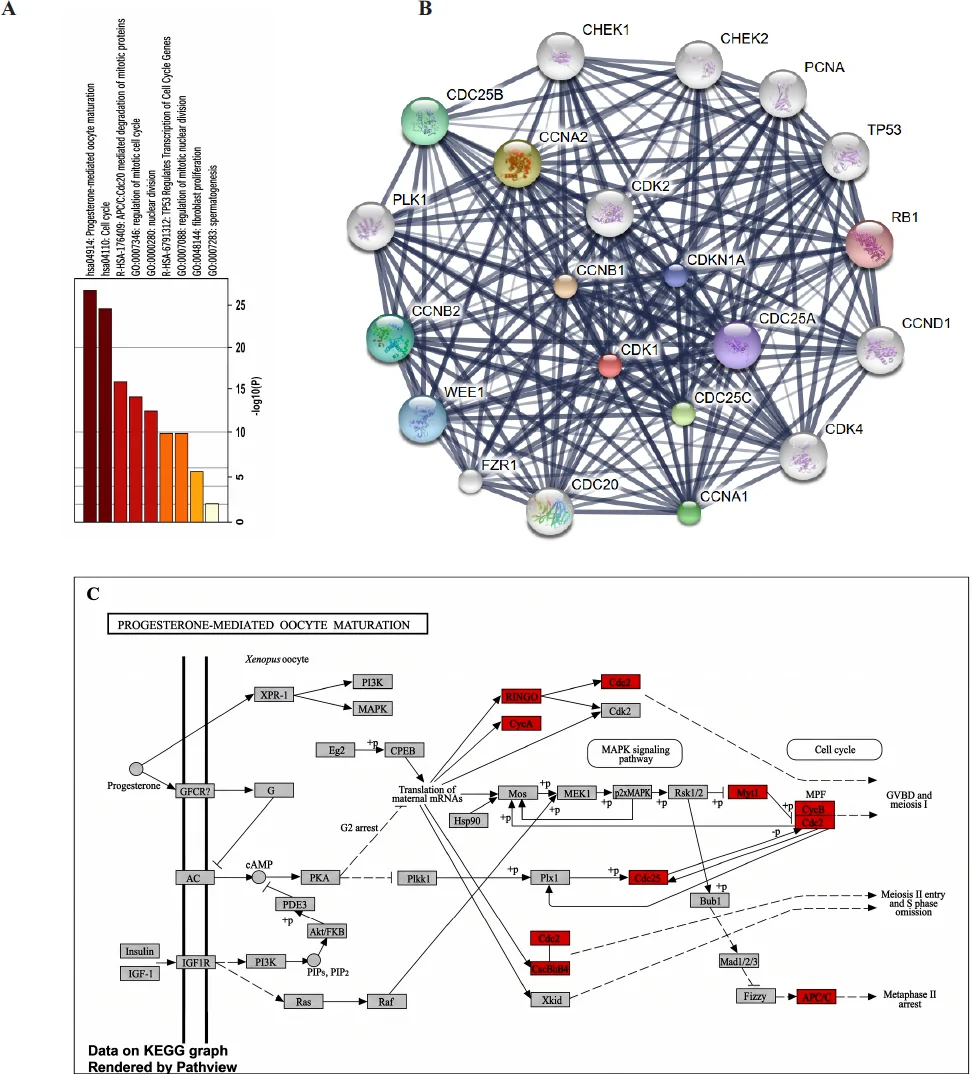
CDK1 signaling pathway and protein interaction analysis (A) Enriched ontology clusters across studies generated by Metascape. (B) An interacting network of CDK1 and its interacting proteins was generated *via* the STRING database. (C) Signaling pathways involving CDK1-interacting proteins through KEGG pathway enrichment analyses using ShinyGO 0.77.

**Fig. (5):**
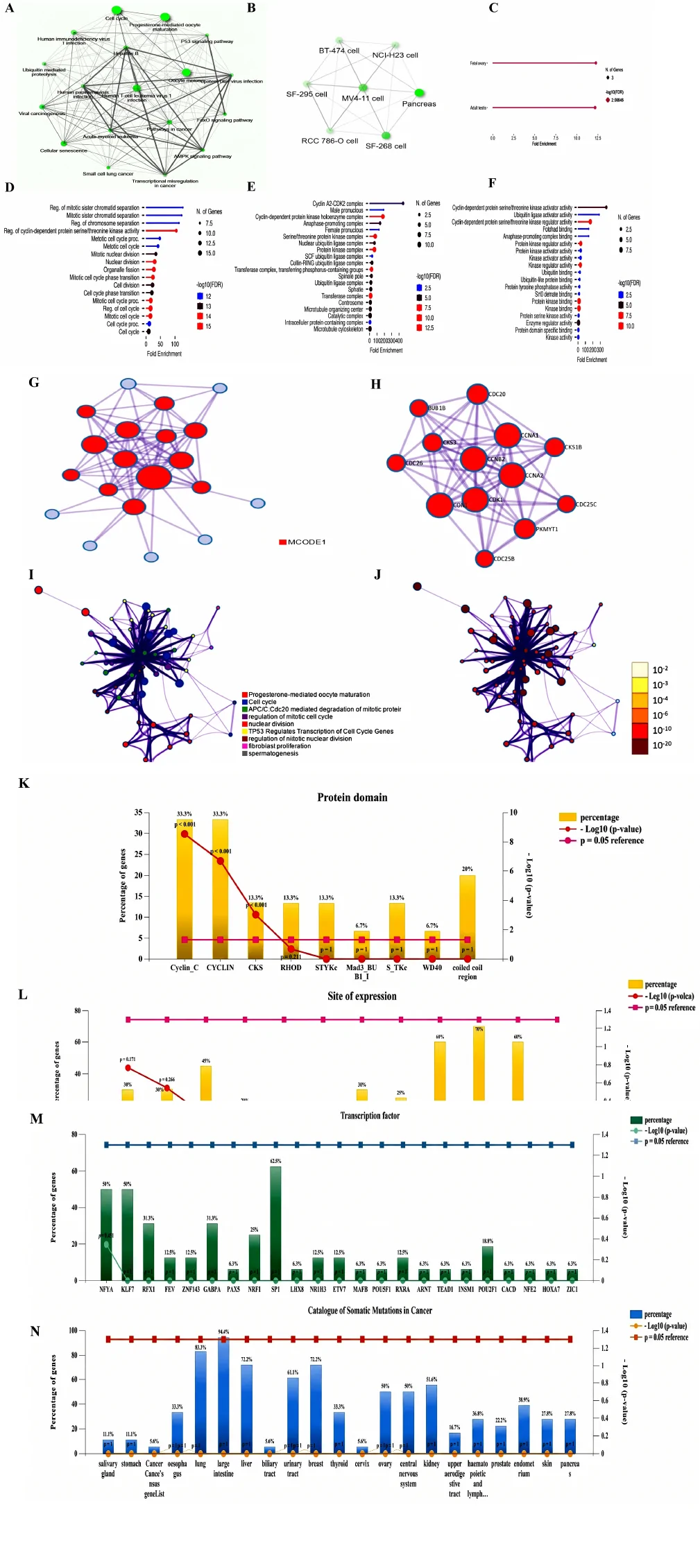
Comprehensive analysis of CDK1 signaling pathways and protein interactions. (A) Signaling pathways: Involvement of CDK1-interacting proteins identified through KEGG pathway enrichment analyses (B). Showing co-expression, tissue-specific, and protein expression profiles. (C) The human proteome's co-expression and tissue-specific protein expression profiles (D) Gene Ontology (GO) Enrichment analyses: Biological Processes. (E) GO Enrichment Analyses: Cellular Components. (F) GO Enrichment Analyses: Molecular Functions. (A-F) were identified through ShinyGO 0.77. (G) Protein-protein interaction network. (H) PPI MCODE Components. (I) Enriched Ontology Clusters (Colored by Cluster ID) (J) Enriched ontology clusters (Colored by p-Value). (G-J) were generated by Metascape. (K) Protein Domain. (L) Site of Expression. (M) Transcription Factors. (N) Catalogue of Somatic Mutations in Cancer. (K-N) analyzed using FunRich.

**Fig. (6):**
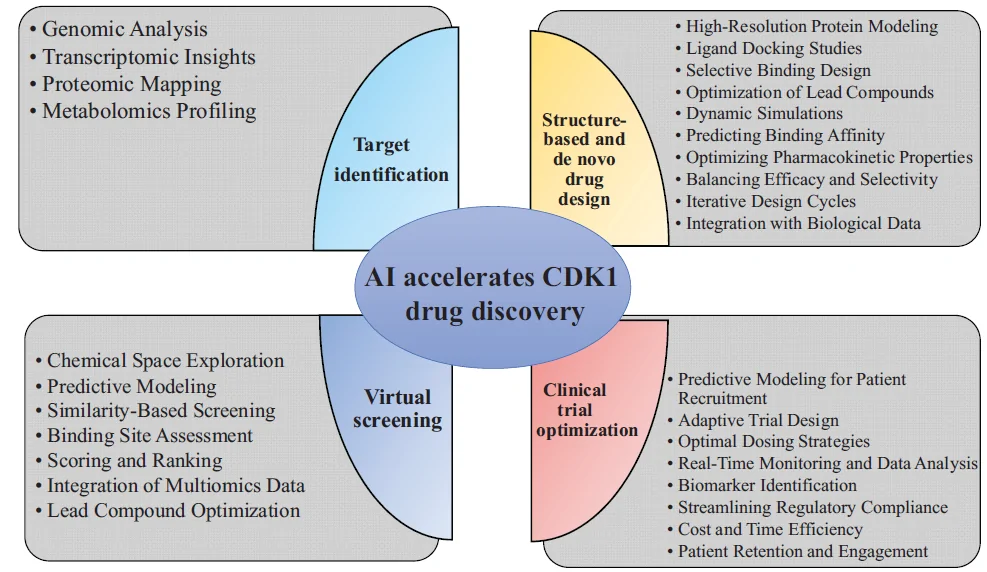
Applications of AI in the development of CDK1 inhibitor drugs, such as target identification, drug optimization, and customized therapy.

**Table 1:** Positive and negative mediators, Inhibitors in clinical and preclinical studies, Oncogenes, Tumor suppressors, and Cell cycle substrates found in different literature.

Positive Mediators [7, 40-42]	Negative Mediators [40, 43]	Inhibitors in Preclinical Studies [7, 44]	CDK1 Activation [45, 46]	CDK1 Expression [47-49]	Inhibitors in Clinical Studies [7, 44]	Oncogenes [6, 7, 50]	Tumor Suppressors [6, 24, 51, 52]	Substrates in Cell Cycle [4, 5]
CDK7	WEE1	RO-3306	Cyclin B1	MYC	BEY1107	BRAF	p53	BORA
KCTD12	CSNK2B	Compound 52	CDC25C	CCNB1	Flavopiridol	AFT7	BORA	LBR
LAR	PP4C	Cgp-74514A	PLK1	E2F1	Ponicidin	MDM2	LBR	TMAP
NOL1	MYT1	Alsterpaullone	CHK1	RBL1	P276-00	ERG3	MLL5	CHK1
MCM10	PRKDC	ATX-313	Aurora A	TP53	Dinaciclib	RUNX1	MLL	RPL12
MRF19	PTEN	Staurosporine	-	-	AT7519	SIRT3	GWL	CDC21A
TGFβ	DYRK1A	JNJ-7706621	-	-	Seliciclib	AR	EML3	TRF1
PFKFB3	DBF4	Olomoucine	-	-	AG-024322	RUNX2	CASP8	CREB
CDC25A	USP7	AT-7519	-	-	PH4-793887	NCOA3	KIF4A	GM130
MASTL	CHEK1	R547	-	-	R547	HIF1A	CLASP2	ELAVL1
CDC25C	P17	Indirubin	-	-	RGB-286638	RXRA	SP1	CENPA
TSPY	IFI27	Indirubin-5-Sulfonate	-	-	Indirubin	EZH2	CASP9	VIM
KPNB1	P21	SU9516	-	-	-	CEBPA	VIP135	CUX1
SIB1	FGF1	AZD5438	-	-	-	YAP	NUMA	NCM3
-	CDK8	Dinaciclib	-	-	-	CEBPA	CM1	EZH2
-	SET	BMS-265246	-	-	-	TCF4	DAPK1	TOP1
-	BUB1	-	-	-	-	AJUBA	BL61	CDC45
-	DUX4	-	-	-	-	TCP2L1	PHE2	PCNA
-	CDH1	-	-	-	-	BCL9	SENP3	TMAP
-	ESR2	-	-	-	-	BCL2	TIF1	XIAP
-	-	-	-	-	-	PRKCB	VGL4	NIPA
-	-	-	-	-	-	BCL2L1	NCL	-

**Table 2:** Some crystal structures of CDK1 with different inhibitors reported in the literature.

**PDB ID**	**Resolution**	**3D Structure with Ligand**	**Ligand**	**2D Structure Shows Residues Forming H-bonds (Green Color) and Hydrophobic Interactions with the Ligand**
5HQ0 (Ternary complex of human proteins CDK1, Cyclin B and CKS2, bound to an inhibitor) [13]	2.30 Å	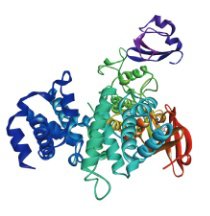	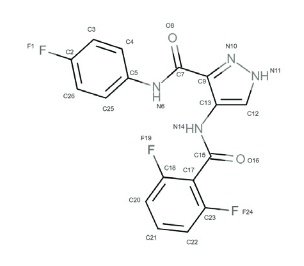	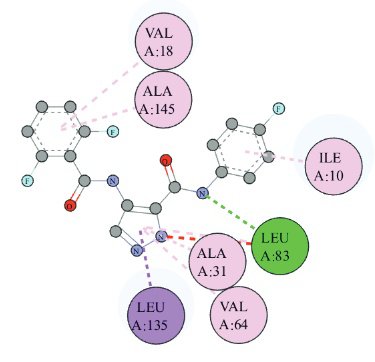
5LQF (CDK1/CyclinB1/CKS2 in complex with NU6102) [68]	2.06 Å	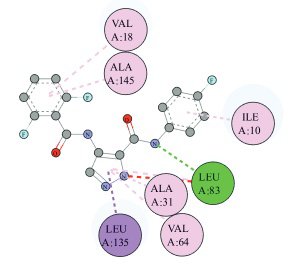	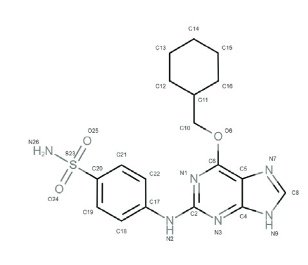	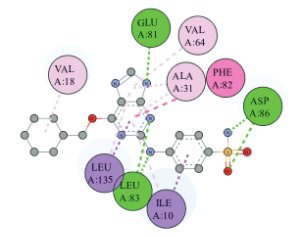
6GU2 (CDK1/CyclinB/Cks2 in complex with Flavopiridol) [69]	2.00 Å	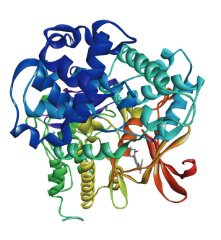	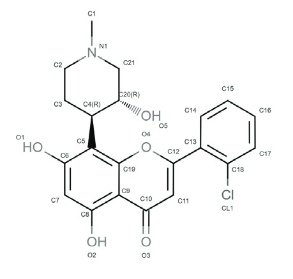	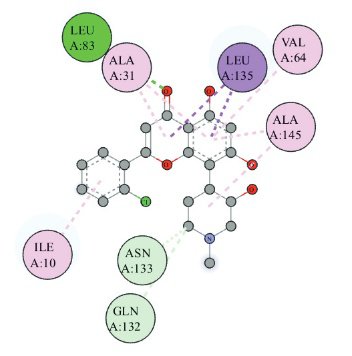
6GU3 (CDK1/CyclinB/Cks2 in complex with AZD5438) [69]	2.65 Å	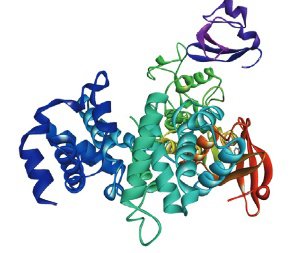	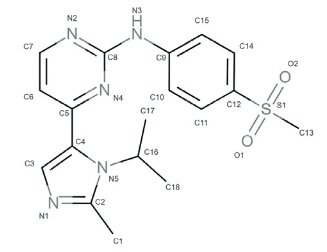	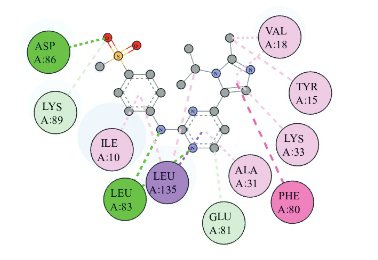
6GU4 (CDK1/CyclinB/Cks2 in complex with CGP74514) [69]	2.73 Å	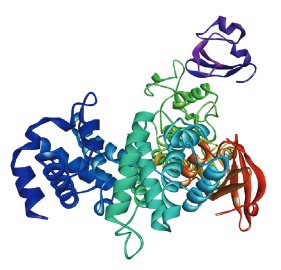	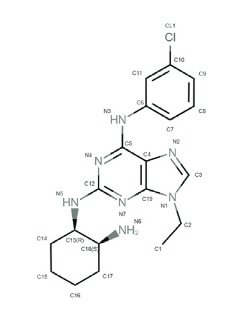	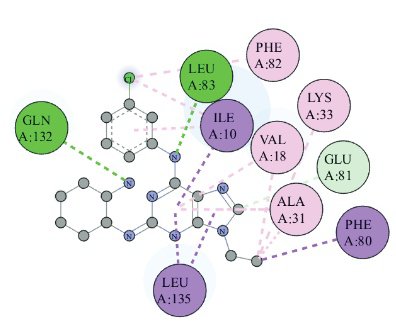
6GU6 (CDK1/Cks2 in complex with Dinaciclib) [69]	2.33 Å	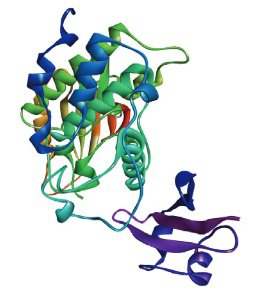	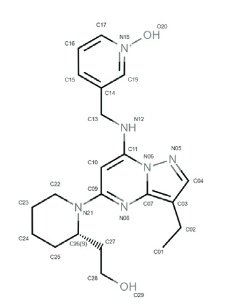	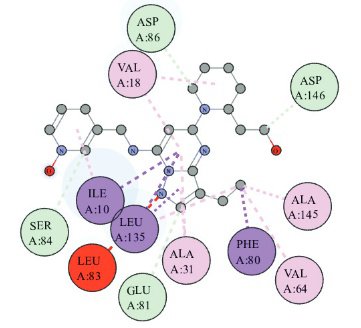

**Table 3:** Small-molecule CDK1 inhibitors reported in the literature.

**Drug Name**	**Structure**	**Major Targets**	**IC_50_ Against CDK1**	**Cancer Type**	**Mechanism in Cancer Cells**
BEY1107 (Avotaciclib) [78]	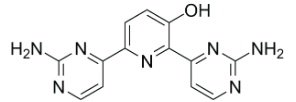	CDK1	2 nM	Pancreatic Cancer, Metastatic colorectal cancer (combination with Capecitabine)	Induces cell cycle arrest and apoptosis, stopping cancer cell growth
Flavopiridol (Alvocidib) [79]	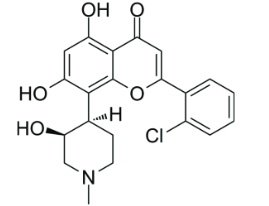	CDK1/2/4/7/9	30 nM	AML, breast cancer, and prostate cancer	Induces cell cycle arrest, triggers apoptosis, and inhibits transcription.
Roniciclib [80]	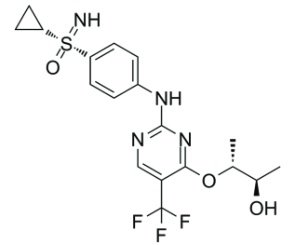	CDK1/2/3/4/7/9	5–25 nM	SCLC	Leads to cell cycle arrest, apoptosis, and inhibition of tumor growth
P276-00 [81]	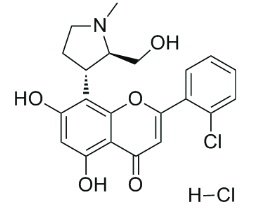	CDK1/4/9	79 nM	Mantle Cell Lymphoma (MCL), pancreatic cancer (combination with gemcitabine)	Induces cell cycle arrest and apoptosis
Dinaciclib [80]	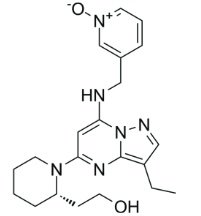	CDK1/2/5/9	3 nM	Chronic Lymphocytic Leukaemia (CLL), MCL, Diffuse Large B-Cell Lymphoma (DLBCL), and solid tumors	Cell cycle arrest and apoptosis
AT7519 [82]	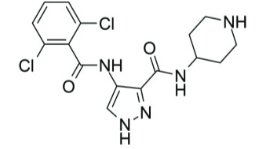	CDK1/2/4/6/9	210 nM	Solid tumors, haematological malignancies, and CLL	Antiproliferative activity by causing cell cycle arrest and apoptosis
Seliciclib (CYC202 or R-roscovitine) [83]	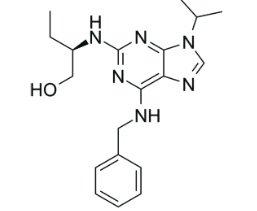	CDK1/2/7/9	86 μM	NSCLC	This leads to cell cycle arrest and apoptosis
AG-024322 [84, 85]	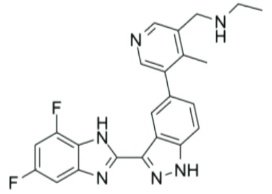	CDK1/2/4	2.3 nM	Solid tumors and haematological malignancies. Multiple human tumor xenograft models, including breast, lung, and colon cancers	Causes cell cycle arrest and apoptosis
PHA-793887 [86, 87]	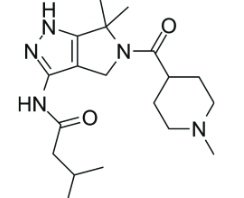	CDK1/2/4/5/7/9	60 nM	Various cancers, including acute leukaemias (such as HL60, K562, ALL-2, and AML-PS) and several solid tumor cell lines	Blocks Rb phosphorylation and S-phase cyclins expression, leading to cell cycle arrest and apoptosis
R547 [88]	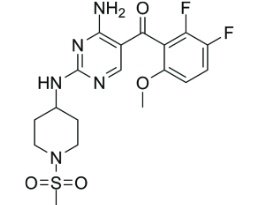	CDK1/2/4/7	2 nM	Solid tumors, including colon, lung, breast, prostate, and melanoma cancer models.	Induce cell cycle arrest at the G1 and G2 phases and promote apoptosis
RGB-286638 [89]	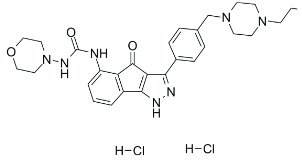	CDK1/2/3/4/5/9	2-5 nM	Various solid tumors and haematological malignancies	Induce cell cycle arrest and apoptosis
AZD-5438 [90]	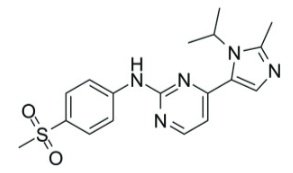	CDK1/2/9	16 nM	Neoplasms	It induces cell cycle arrest at the G2-M, S, and G1 phases and inhibits the phosphorylation of CDK substrates, leading to antiproliferative effects
Indirubin (Natural compound from the indigo plant) [91]	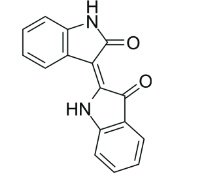	CDK1/2/4/5	180 nM	Small-cell lung cancer, glioblastoma, and chronic myeloid leukaemia	Inhibits Glycogen Synthase Kinase-3 (GSK-3), leading to cell cycle arrest and apoptosis

**Table 4:** Dysregulated expression and functions of the CDK1 signaling pathway proteins contribute to the intrinsic resistance to CDK1 inhibitors.

**Protein**	**Expression**	**Type of Cancer**	**Role**	**Specific CDK1 Inhibitors**
CCND1 [92]	Overexpression of Cyclin D1	Endometrial cancer	Enhances the sensitivity to CDK1 and other CDK inhibitors	RO-3306, AZD5438
CCNE1 [93]	Overexpression of Cyclin E1	HR+ HER2- breast cancer	Resistance to CDK1 and other CDK inhibitors	RO-3306, Flavopiridol
CDK2 [93]	Increased CDK2 activity	Luminal androgen receptor breast cancer	Resistance to CDK1 and other CDK inhibitors	AZD5438, Alsterpaullone
CDK4 [94]	CDK4-amplification and activation, T172 phosphorylation	Liposarcoma, Neuroblastoma, Breast cancer	Sensitivity to CDK1 and other CDK inhibitors enhances sensitivity to CDK1 inhibitors in breast cancer	RO-3306, Flavopiridol
CDKN2A [95]	p16INK4A loss	Melanoma, Pancreatic ductal adenocarcinoma, Glioblastoma	Sensitivity to CDK1 inhibitors in melanoma, resistance in pancreatic ductal adenocarcinoma, susceptibility in glioblastoma	Purvalanol A, Roscovitine
E2F [96]	Activation of E2F	Mutant BRAF and NRAS melanoma	Resistance to CDK1 and other CDK inhibitors	SNS-032, Alsterpaullone
E2F4 [97]	Activation of E2F4	ER+ breast cancer	Resistance to CDK1 inhibitors	AZD5438, Roscovitine
Rb1 [98]	Rb1 loss and degradation	Breast, ovarian, HPV-positive cervical, head and neck cancer	Resistance to CDK1 inhibitors in breast and ovarian cancer, and in HPV-positive cervical and head and neck cancer	BMS-265246, RO-3306
TP53 [24]	p53 loss and degradation	Breast cancer	Resistance to CDK1 and other CDK inhibitors	Dinaciclib, RO-3306

**Table 5:** Overview of advanced clinical trials of selected 1st, 2nd, and 3rd generation CDK1 inhibitors in cancer therapy.

**Generation**	**CDKI**	**Targeted CDKs**	**Clinical Trial Phases**	**Immediate Implications in Cancer Therapy**	**Future Technologies**
1^st^	Flavopiridol [107]	CDK1, CDK2, CDK4, CDK6	Phase I, II	Combination therapies to reduce toxicity and enhance efficacy	CRISPR/Cas9 Technology, Nanotechnology, Artificial intelligence and machine learning, Combination therapies, and biomarker identification
	Roscovitine [44]	CDK1, CDK2, CDK5, CDK7, CDK9	Phase I, II	Combination with other treatments to improve outcomes	CRISPR gene editing, Nanotechnology, AI-driven drug discovery
2^nd^	Dinaciclib [108]	CDK1, CDK2, CDK5, CDK9	Phase I, II, III	Combination with immunotherapy and targeted therapies	CRISPR gene editing, Nanotechnology, Artificial intelligence and machine learning, and biomarker identification
	AT7519 [109]	CDK1, CDK2, CDK4, CDK5, CDK9	Phase I, II	Combination with other targeted therapies to enhance efficacy	CRISPR gene editing, Nanotechnology, Artificial intelligence, and machine learning,
3^rd^	Palbociclib [110]	CDK1, CDK4, CDK6	Phase I, II, III	Combination with other targeted therapies and immunotherapies	CRISPR/Cas9 Technology, Nanotechnology, AI-driven drug discovery
	Ribociclib [111]	CDK1, CDK4, CDK6	Phase I, II, III	Combination with other targeted therapies and immunotherapies	CRISPR/Cas9 Technology, Nanotechnology, AI-driven drug discovery, and combination therapies

## LIST OF ABBREVIATIONS

AI: Artificial Intelligence
APC/C: Anaphase-Promoting Complex/Cyclosome
CDKs: Cyclin-Dependent Kinases
GO: Gene Ontology
KEGG: Kyoto Encyclopedia of Genes and Genomes

## References

[1] Malumbres M. (2014). Cyclin-dependent kinases.. Genome Biol..

[2] Łukasik P., Załuski M., Gutowska I. (2021). Cyclin-dependent kinases (CDK) and their role in diseases development-review.. Int. J. Mol. Sci..

[3] Porter L.A., Donoghue D.J. (2003). Cyclin B1 and CDK1: Nuclear localization and upstream regulators.. Prog. Cell. Cycle Res..

[4] Massacci G., Perfetto L., Sacco F. (2023). The cyclin-dependent kinase 1: More than a cell cycle regulator.. Br. J. Cancer.

[5] Enserink J.M., Kolodner R.D. (2010). An overview of Cdk1-controlled targets and processes.. Cell. Div..

[6] Ghafouri-Fard S., Khoshbakht T., Hussen B.M., Dong P., Gassler N., Taheri M., Baniahmad A., Dilmaghani N.A. (2022). A review on the role of cyclin dependent kinases in cancers.. Cancer Cell. Int..

[7] Wang Q., Bode A.M., Zhang T. (2023). Targeting CDK1 in cancer: Mechanisms and implications.. NPJ. Precis. Oncol..

[8] Łukasik P., Baranowska-Bosiacka I., Kulczycka K., Gutowska I. (2021). Inhibitors of cyclin-dependent kinases: Types and their mechanism of action.. Int. J. Mol. Sci..

[9] Sofi S., Mehraj U., Qayoom H., Aisha S., Almilaibary A., Alkhanani M., Mir M.A. (2022). Targeting cyclin-dependent kinase 1 (CDK1) in cancer: Molecular docking and dynamic simulations of potential CDK1 inhibitors.. Med. Oncol..

[10] Vishwakarma K.K., Kolthur-Seetharam U., Venkatramani R. (2024). ATP-binding free energy simulations reveal an allosteric link between the enzyme active site and multiple functional protein-protein interaction interfaces in cyclin dependent kinase-1.. bioRxiv.

[11] Recabarren R., Osorio E.H., Caballero J., Tuñón I., Alzate-Morales J.H. (2019). Mechanistic insights into the phosphoryl transfer reaction in cyclin-dependent kinase 2: A QM/MM study.. PLoS One.

[12] Rath S.L., Senapati S. (2013). Molecular basis of differential selectivity of cyclobutyl-substituted imidazole inhibitors against CDKs: insights for rational drug design.. PLoS One.

[13] Brown N.R., Korolchuk S., Martin M.P., Stanley W.A., Moukhametzianov R., Noble M.E.M., Endicott J.A. (2015). CDK1 structures reveal conserved and. unique features of the essential cell cycle CDK. Nat. Commun.

[14] Into Evolution S.P.I. (2005). Global analysis of cdk1 substrate phosphorylation.. J. Biol. Chem..

[15] Miguel R.B., Petersen P.A.D., Gonzales-Zubiate F.A., Oliveira C.C. (2015). Kumar, N.; do Nascimento, R.R.; Petrilli, H.M.; da Costa Ferreira, A.M. Inhibition of cyclin-dependent kinase CDK1 by oxindolimine ligands and corresponding copper and zinc complexes.. J. Biol. Inorg. Chem..

[16] Kachroo A.H., Vandeloo M., Greco B.M., Abdullah M. (2022). Humanized yeast to model human biology, disease and evolution.. Dis. Model Mech..

[17] Fisher R.P. (2005). Secrets of a double agent: CDK7 in cell-cycle control and transcription.. J. Cell. Sci..

[18] Lemonnier T., Dupré A., Jessus C. (2020). The G2-to-M transition from a phosphatase perspective: a new vision of the meiotic division.. Cell. Div.

[19] Fourest-Lieuvin A., Peris L., Gache V., Garcia-Saez I., Juillan-Binard C., Lantez V., Job D. (2006). Microtubule regulation in mitosis:. Tubulin phosphorylation by the cyclin-dependent kinase Cdk1. Mol. Biol. Cell..

[20] Li Z., Tian X., Ji X., Wang J., Chen H., Wang D., Zhang X. (2020). ULK1-ATG13 and their mitotic phospho-regulation by CDK1 connect autophagy to cell cycle.. PLoS. Biol..

[21] Ravindran Menon D., Luo Y., Arcaroli J.J., Liu S., KrishnanKutty L.N., Osborne D.G., Li Y., Samson J.M., Bagby S., Tan A.C., Robinson W.A., Messersmith W.A., Fujita M. (2018). CDK1 interacts with Sox2 and promotes tumor initiation in human melanoma.. Cancer Res..

[22] Preisinger C., Körner R., Wind M., Lehmann W.D., Kopajtich R., Barr F.A. (2005). Plk1 docking to GRASP65 phosphorylated by Cdk1 suggests a mechanism for Golgi checkpoint signalling.. EMBO J..

[23] Liu X., Wu H., Liu Z. (2022). An integrative human pan-cancer analysis of cyclin-dependent kinase 1 (CDK1).. Cancers.

[24] Mir M.A., Haq B.U., Mir M. (2023). CDK1 dysregulation in breast cancer.. Therapeutic potential of cell cycle kinases in breast cancer.

[25] Han Z., Jia Q., Zhang J., Chen M., Wang L., Tong K., He W., Zhang Y., Zhu W., Qin J., Wang T., Liu T., Ma Y., Chen Y., Zha S., Zhang C. (2023). Deubiquitylase YOD1 regulates CDK1 stability and drives triple-negative breast cancer tumorigenesis.. J. Exp. Clin. Cancer Res..

[26] Karlsson C., Katich S., Hagting A., Hoffmann I., Pines J. (1999). Cdc25B and Cdc25C differ markedly in their properties as initiators of mitosis.. J. Cell. Biol..

[27] Bukhari A.B., Chan G.K., Gamper A.M. (2022). Targeting the DNA damage response. for cancer therapy by inhibiting the kinase Wee1. Front Oncol..

[28] Zitouni S., Francia M.E., Leal F., Montenegro Gouveia S., Nabais C., Duarte P., Gilberto S., Brito D., Moyer T., Kandels-Lewis S., Ohta M., Kitagawa D., Holland A.J., Karsenti E., Lorca T., Lince-Faria M., Bettencourt-Dias M. (2016). CDK1 prevents unscheduled PLK4-STIL complex assembly in centriole biogenesis.. Curr. Biol..

[29] Saavedra K., Valbuena J., Olivares W., Marchant M.J., Rodríguez A., Torres-Estay V., Carrasco-Avino G., Guzmán L., Aguayo F., Roa J.C., Corvalán A.H. (2015). Loss of expression of reprimo, a p53-induced cell cycle arrest gene, correlates with invasive stage of tumor progression and p73 expression in gastric cancer.. PLoS One.

[30] Wang H., Dong W., Wang L. (2019). Expression profile and potential functional differentiation of the Speedy/RINGO family in mice.. Gene.

[31] Marjanović M., Sánchez-Huertas C., Terré B., Gómez R., Scheel J.F., Pacheco S., Knobel P.A., Martínez-Marchal A., Aivio S., Palenzuela L., Wolfrum U., McKinnon P.J., Suja J.A., Roig I., Costanzo V., Lüders J., Stracker T.H. (2015). CEP63 deficiency promotes p53-dependent microcephaly and reveals a role for the centrosome in meiotic recombination.. Nat. Commun..

[32] Hégarat N., Vesely C., Vinod P.K., Ocasio C., Peter N., Gannon J., Oliver A.W., Novák B., Hochegger H. (2014). PP2A/B55 and Fcp1 regulate Greatwall and Ensa dephosphorylation during mitotic exit.. PLoS Genet..

[33] Schmit F., Cremer S., Gaubatz S. (2009). LIN54 is an essential core subunit of the DREAM/LINC complex that binds to the cdc2 promoter in a sequence-specific manner.. FEBS J..

[34] Rogers S., Fey D., McCloy R.A., Parker B.L., Mitchell N.J., Payne R.J., Daly R.J., James D.E., Caldon C.E., Watkins D.N., Croucher D.R., Burgess A. (2016). PP1 initiates the dephosphorylation of MASTL, triggering mitotic exit and bistability in human cells.. J. Cell. Sci..

[35] Pecoraro C (2024). The Role of speedy/ringo in triple negative breast cancer metastasis... University of Windsor.

[36] Counts S.E., Mufson E.J. (2017). Regulator of cell cycle (RGCC) expression during the progression of Alzheimer's disease.. Cell. Transplant..

[37] Hung K.S., Hsiao C.C., Pai T.W., Hu C.H., Tzou W.S., Wang W.D., Chen Y.R. (2018). Functional enrichment analysis based on long noncoding RNA associations.. BMC Syst. Biol..

[38] Doncheva N.T., Morris J.H., Gorodkin J., Jensen L.J. (2019). Cytoscape StringApp: Network analysis and visualization of proteomics data.. J. Proteome Res..

[39] Bader G.D., Hogue C.W.V. (2003). An automated method for finding molecular complexes in large protein interaction networks.. BMC Bioinformatics.

[40] Yu B., Dalton W.B., Yang V.W. (2012). CDK1 regulates mediator of DNA damage checkpoint 1 during mitotic DNA damage.. Cancer Res..

[41] Zhong Y., Yang J., Xu W.W., Wang Y. (2017). Zheng, C-C.; Li, B.; He, Q-Y. KCTD12 promotes tumorigenesis by facilitating CDC25B/CDK1/Aurora A-dependent G2/M transition.. Oncogene.

[42] Yamakawa H., Ebara S., Yoshida M., Yamamoto K., Hayashi K., Shimokawa K., Araki S., Tauchi H., Morishita D. (2024). Phosphorylation dynamics and kinase interaction in mRNA transcription-associated kinases: autophosphorylation of. CDK12 and its modulation by CDK7 and CDK9. BPB Reports.

[43] Fei Q., Zou Z., Roundtree I.A., Sun H.L., He C. (2020). YTHDF2 promotes mitotic entry and is regulated by cell cycle mediators.. PLoS Biol..

[44] Cicenas J., Valius M. (2011). The CDK inhibitors in cancer research and therapy.. J. Cancer Res. Clin. Oncol..

[45] Liu K., Zheng M., Lu R., Du J., Zhao Q., Li Z., Li Y., Zhang S. (2020). The role of CDC25C in cell cycle regulation and clinical cancer therapy: A systematic review.. Cancer Cell. Int..

[46] Liu K., Zheng M., Zhao Q., Zhang K., Li Z., Fu F., Zhang H., Du J., Li Y., Zhang S. (2020). Different p53 genotypes regulating different phosphorylation sites and subcellular location of CDC25C associated with the formation of polyploid giant cancer cells.. J. Exp. Clin. Cancer Res..

[47] Huang Y., Li D., Wang L., Su X., Tang X. (2022). CENPF/CDK1 signaling pathway enhances the progression of adrenocortical carcinoma by regulating the G2/M-phase cell cycle.. J. Transl. Med..

[48] Yang Y., Xue K., Li Z., Zheng W., Dong W., Song J., Sun S., Ma T. (2018). Li, W. c-Myc regulates the CDK1/cyclin B1 dependent-G2/M cell cycle progression by histone H4 acetylation in Raji cells.. Int. J. Mol. Med..

[49] Kang J., Sergio C.M., Sutherland R.L., Musgrove E.A. (2014). Targeting cyclin-dependent kinase 1 (CDK1) but not CDK4/6 or CDK2 is selectively lethal to MYC-dependent human breast cancer cells.. BMC Cancer.

[50] Talatam A., Reddy P.K., Motohashi N., Vanam A., Gollapudi R. (2023). Targeting overexpressed cyclin dependent kinase 1 (CDK1) in human cancers: Kamalachalcone A emerged as potential inhibitor of CDK1 kinase through in silico docking study.. Oncogen.

[51] Goga A., Yang D., Tward A.D., Morgan D.O., Bishop J.M. (2007). Inhibition of CDK1. as a potential therapy for tumors over-expressing MYC. Nat. Med..

[52] Mir M. (2023). Mir, M Khan, S. Aisha, S. Therapeutic potential of cell cycle kinases in breast cancer.

[53] Zhang C., Elkahloun A.G., Robertson M., Gills J.J., Tsurutani J., Shih J.H., Fukuoka J., Hollander M.C., Harris C.C., Travis W.D., Jen J., Dennis P.A. (2011). Loss of cytoplasmic CDK1 predicts poor survival in human lung cancer and confers chemotherapeutic resistance.. PLoS One.

[54] Radomska H.S., Alberich-Jordà M., Will B., Gonzalez D., Delwel R., Tenen D.G. (2012). Targeting CDK1 promotes. FLT3-activated acute myeloid leukemia differentiation through C/EBPα. J. Clin. Invest.

[55] Thoma O.M., Neurath M.F., Waldner M.J. (2021). Cyclin-dependent kinase inhibitors and their therapeutic potential in colorectal cancer treatment.. Front. Pharmacol..

[56] Serpico A.F., Grieco D. (2020). Recent advances in understanding the. role of CDK1 in the Spindle Assembly Checkpoint. F1000 Res..

[57] Androic I., Krämer A., Yan R., Rödel F., Gätje R., Kaufmann M., Strebhardt K., Yuan J. (2008). Targeting cyclin B1 inhibits proliferation and sensitizes breast cancer cells to taxol.. BMC Cancer.

[58] Karn V., Sandhya S., Hsu W., Parashar D., Singh H.N., Jha N.K., Gupta S., Dubey N.K., Kumar S. (2022). CRISPR/Cas9 system in breast cancer therapy: Advancement, limitations and future scope.. Cancer Cell. Int..

[59] Ma J., Xue K., Jiang Y., Wang X., He D., Guo P. (2024). Down-regulation of SLC14A1 in prostate cancer activates CDK1/CCNB1 and mTOR pathways and promotes tumor progression.. Sci. Rep.

[60] Chen, S Xu, Y. Yuan, X. Bubley, G.J. Balk, S.P. Androgen receptor phosphorylation and stabilization in prostate cancer by cyclin-dependent kinase 1. Proc. Natl. Acad. Sci. USA, 2006, 103(43), 15969-15974.

[61] Voce D.J., Bernal G.M., Cahill K.E., Wu L., Mansour N., Crawley C.D., Campbell P.A.S., Arina A., Weichselbaum R.R., Yamini B. (2021). CDK1 is up-regulated by temozolomide in an NF-κB dependent manner in glioblastoma.. Sci. Rep..

[62] Han K., Chen Y., Sun X., Wen L., Wu Y., Chen S., Wei L., Yu J., Zeng T., Jiang L., Tan L. (2024). Combining serum CDK1 with tumor markers for the diagnosis of small cell lung cancer.. Clin. Transl. Oncol..

[63] Du Q., Liu W., Mei T., Wang J., Qin T., Huang D. (2023). Prognostic and immunological characteristics of CDK1 in lung adenocarcinoma: A systematic analysis.. Front Oncol..

[64] Bencivenga D., Stampone E., Azhar J., Parente D., Ali W., Del Vecchio V., Della Ragione F. (2025). Borriello, A. p27Kip1 and Tumors: Characterization. of CDKN1B Variants Identified in MEN4 and Breast Cancer. Cells.

[65] Wijnen R., Pecoraro C., Carbone D., Fiuji H., Avan A., Peters G.J., Giovannetti E., Diana P. (2021). Cyclin dependent kinase-1 (CDK-1) inhibition as. a novel therapeutic strategy against pancreatic ductal adenocarcinoma (PDAC). Cancers.

[66] Dhir T., Schultz C.W., Jain A., Brown S.Z., Haber A., Goetz A., Xi C., Su G.H., Xu L., Posey J., Jiang W., Yeo C.J., Golan T., Pishvaian M.J., Brody J.R. (2019). Abemaciclib is effective against pancreatic cancer cells and synergizes with HuR and YAP1 inhibition.. Mol. Cancer Res..

[67] Johnson N., Li Y.C., Walton Z.E., Cheng K.A., Li D., Rodig S.J., Moreau L.A., Unitt C., Bronson R.T., Thomas H.D., Newell D.R., D'Andrea A.D., Curtin N.J., Wong K.K., Shapiro G.I. (2011). Compromised CDK1 activity sensitizes BRCA-proficient cancers to PARP inhibition.. Nat. Med..

[68] Coxon C.R., Anscombe E., Harnor S.J., Martin M.P., Carbain B., Golding B.T., Hardcastle I.R., Harlow L.K., Korolchuk S., Matheson C.J., Newell D.R., Noble M.E.M., Sivaprakasam M., Tudhope S.J., Turner D.M., Wang L.Z., Wedge S.R., Wong C., Griffin R.J., Endicott J.A., Cano C. (2017). Cyclin-dependent kinase (CDK) inhibitors: Structure-activity relationships and insights into the CDK-2 selectivity of 6-substituted 2-arylaminopurines.. J. Med. Chem..

[69] Wood D.J., Korolchuk S., Tatum N.J., Wang L.-Z., Endicott J.A., Noble M.E., Martin M.P. (2019). Differences in the conformational energy landscape of CDK1 and CDK2 suggest a mechanism for achieving selective CDK inhibition.. Cell. Chem. Biol..

[70] Korolchuk S.M (2018). Structural and functional characterisation of cyclin dependent kinase 1 containing complexes... Newcastle University.

[71] Holder J., Mohammed S., Barr F.A. (2020). Ordered dephosphorylation initiated by the selective proteolysis of cyclin B drives mitotic exit.. eLife.

[72] Yu J., Raia P., Ghent C.M., Raisch T., Sadian Y., Cavadini S., Sabale P.M., Barford D., Raunser S., Morgan D.O., Boland A. (2021). Structural basis. of human separase regulation by securin and CDK1-cyclin B1. Nature.

[73] Gough R.E., Jones M.C., Zacharchenko T., Le S., Yu M., Jacquemet G., Muench S.P., Yan J., Humphries J.D., Jørgensen C., Humphries M.J., Goult B.T. (2021). Talin mechanosensitivity is modulated by a direct interaction with cyclin-dependent kinase. J. Biol. Chem..

[74] Wood D.J., Endicott J.A. (2018). Structural insights into the functional diversity of the CDK-cyclin family.. Open Biol..

[75] Brown N.R., Noble M.E.M., Endicott J.A., Johnson L.N. (1999). The structural basis for specificity of substrate and recruitment peptides for cyclin-dependent kinases.. Nat. Cell. Biol..

[76] Aaltonen K. (2009). Amini, R-M.; Heikkilä, P.; Aittomäki, K.; Tamminen, A.; Nevanlinna, H.; Blomqvist, C. High cyclin B1 expression is associated with poor survival in breast cancer.. Br. J. Cancer.

[77] Hope I., Endicott J.A., Watt J.E. (2023). Emerging approaches to CDK inhibitor development, a structural perspective.. RSC. Chem. Biol..

[78] Garcia-Sampedro A., Gaggia G., Ney A., Mahamed I., Acedo P. (2021). The state-of-the-art of phase II/III clinical trials for targeted pancreatic cancer therapies.. J. Clin. Med..

[79] Baghel U.S., Kriplani P., Patel N.M., Kaur M., Sharma K., Meghani M., Sharma A., Singh D., Singh B., Setzer W.N., Sharifi-Rad J., Calina D. (2025). Flavopiridol: A promising cyclin-dependent kinase inhibitor in cancer treatment.. Naunyn Schmiedebergs Arch. Pharmacol..

[80] Bahleda R., Grilley-Olson J.E., Govindan R., Barlesi F., Greillier L., Perol M., Ray-Coquard I., Strumberg D., Schultheis B., Dy G.K., Zalcman G., Weiss G.J., Walter A.O., Kornacker M., Rajagopalan P., Henderson D., Nogai H., Ocker M., Soria J.C. (2017). Phase I dose-escalation studies of roniciclib, a pan-cyclin-dependent kinase inhibitor, in advanced malignancies.. Br. J. Cancer.

[81] Shirsath N.P., Manohar S.M., Joshi K.S. (2012). P276-00, a cyclin-dependent kinase inhibitor, modulates cell cycle and induces apoptosis in vitro and in vivo in mantle cell lymphoma cell lines.. Mol. Cancer.

[82] Santo L., Vallet S., Hideshima T., Cirstea D., Ikeda H., Pozzi S., Patel K., Okawa Y., Gorgun G., Perrone G., Calabrese E., Yule M., Squires M., Ladetto M., Boccadoro M., Richardson P.G., Munshi N.C., Anderson K.C., Raje N. (2010). AT7519, A novel small molecule multi-cyclin-dependent kinase inhibitor, induces apoptosis in multiple myeloma via GSK-3β activation and RNA polymerase II inhibition.. Oncogene.

[83] Raje N., Kumar S., Hideshima T., Roccaro A., Ishitsuka K., Yasui H., Shiraishi N., Chauhan D., Munshi N.C., Green S.R., Anderson K.C. (2005). Seliciclib (CYC202 or R-roscovitine), a small-molecule cyclin-dependent kinase inhibitor, mediates activity via down-regulation of Mcl-1 in multiple myeloma.. Blood.

[84] Zhong W.Z., Lalovic B., Zhan J. (2009). Characterization of in vitro and in. vivo metabolism of AG-024322, a novel cyclin-dependent kinase (CDK). Health..

[85] Brown A.P., Courtney C.L., Criswell K.A., Holliman C.L., Evering W., Jessen B.A. (2008). Toxicity and toxicokinetics of the cyclin-dependent kinase inhibitor AG-024322 in cynomolgus monkeys following intravenous infusion.. Cancer Chemother. Pharmacol..

[86] Shaikh J., Patel K., Khan T. (2022). Advances in pyrazole based scaffold as cyclin-dependent kinase 2 inhibitors for the treatment of cancer.. Mini Rev. Med. Chem..

[87] Li T., Weng T., Zuo M., Wei Z., Chen M., Li Z. (2016). Recent progress of cyclin-dependent kinase inhibitors as potential anticancer agents.. Future Med. Chem..

[88] DePinto W., Chu X.J., Yin X., Smith M., Packman K., Goelzer P., Lovey A., Chen Y., Qian H., Hamid R., Xiang Q., Tovar C., Blain R., Nevins T., Higgins B., Luistro L., Kolinsky K., Felix B., Hussain S., Heimbrook D. (2006). In vitro and in vivo activity of R547: A potent and selective cyclin-dependent kinase inhibitor currently in phase I clinical trials.. Mol. Cancer Ther..

[89] van der Biessen D.A.J., Burger H., de Bruijn P., Lamers C.H.J., Naus N., Loferer H., Wiemer E.A.C., Mathijssen R.H.J., de Jonge M.J.A. (2014). Phase I study of RGB-286638, a novel, multitargeted cyclin-dependent kinase inhibitor in patients with solid tumors.. Clin. Cancer Res..

[90] Byth K.F., Thomas A., Hughes G., Forder C., McGregor A., Geh C., Oakes S., Green C., Walker M., Newcombe N., Green S., Growcott J., Barker A., Wilkinson R.W. (2009). AZD5438, a potent oral inhibitor of cyclin-dependent kinases 1, 2, and 9, leads to pharmacodynamic changes and potent antitumor effects in human tumor xenografts.. Mol. Cancer Ther..

[91] Marko D., Schätzle S., Friedel A., Genzlinger A., Zankl H., Meijer L., Eisenbrand G. (2001). Inhibition of cyclin-dependent kinase 1 (CDK1) by indirubin derivatives in human tumour cells.. Br J. Cancer.

[92] Xu J., Lin D.I. (2018). Oncogenic c-terminal cyclin D1 (CCND1) mutations are enriched in endometrioid endometrial adenocarcinomas.. PLoS One.

[93] Guerrero-Zotano Á., Belli S., Zielinski C., Gil-Gil M., Fernandez-Serra A., Ruiz-Borrego M., Ciruelos Gil E.M., Pascual J., Muñoz-Mateu M., Bermejo B., Margeli Vila M., Antón A., Murillo L., Nissenbaum B., Liu Y., Herranz J., Fernández-García D., Caballero R., López-Guerrero J.A., Bianco R., Formisano L., Turner N., Martín M. (2023). CCNE1 and PLK1 mediate resistance to palbociclib in HR+/HER2- metastatic breast cancer.. Clin. Cancer Res..

[94] Bisteau X., Paternot S., Colleoni B., Ecker K., Coulonval K., De Groote P., Declercq W., Hengst L., Roger P.P. (2013). CDK4 T172 phosphorylation is central in a CDK7-dependent bidirectional CDK4/CDK2 interplay mediated by p21 phosphorylation at the restriction point.. PLoS Genet..

[95] Lin J.C., Liu T.P., Yang P.M. (2020). CDKN2A-inactivated pancreatic ductal adenocarcinoma exhibits therapeutic sensitivity to paclitaxel: a bioinformatics study.. J. Clin. Med..

[96] Vachtenheim J., Ondrušová L. (2021). Many distinct ways lead to drug resistance in BRAF-and NRAS-mutated melanomas.. Life..

[97] Guerrero-Zotano A.L., Stricker T.P., Formisano L., Hutchinson K.E., Stover D.G., Lee K.M., Schwarz L.J., Giltnane J.M., Estrada M.V., Jansen V.M., Servetto A., Gavilá J., Perez-Fidalgo J.A., Lluch A., Llombart-Cussac A., Bayar M.A., Michiels S., André F., Arnedos M., Guillem V., Ruiz-Simon A., Arteaga C.L. (2018). ER+ breast cancers resistant to prolonged neoadjuvant letrozole exhibit an E2F4 transcriptional program sensitive to CDK4/6 inhibitors.. Clin. Cancer Res..

[98] Linn P., Kohno S., Sheng J., Kulathunga N., Yu H., Zhang Z., Voon D., Watanabe Y., Takahashi C. (2021). Targeting RB1 loss in cancers.. Cancers.

[99] Hawash M. (2024). Advances in cancer therapy: A comprehensive review of CDK and EGFR inhibitors.. Cells.

[100] Khan S.U., Fatima K., Aisha S., Malik F. (2024). Unveiling the mechanisms and challenges of cancer drug resistance.. Cell. Commun. Signal..

[101] Tetsu O., McCormick F. (2003). Proliferation of cancer cells despite CDK2 inhibition.. Cancer Cell..

[102] Yang Q., Ferrell J.E. (2013). The CDK1-APC/C cell cycle oscillator circuit functions as a time-delayed, ultrasensitive switch.. Nat. Cell. Biol..

[103] Gomatou G., Trontzas I., Ioannou S., Drizou M., Syrigos N., Kotteas E. (2021). Mechanisms of resistance to cyclin-dependent kinase 4/6 inhibitors.. Mol. Biol. Rep.

[104] Panagiotou E., Gomatou G., Trontzas I.P., Syrigos N., Kotteas E. (2022). Cyclin-dependent kinase (CDK) inhibitors in solid tumors: A review of clinical trials.. Clin. Transl. Oncol..

[105] Sokhi S., Lewis C.W., Bukhari A.B., Hadfield J., Xiao E.J., Fung J., Yoon Y.J., Hsu W.H., Gamper A.M., Chan G.K. (2023). Myt1 overexpression mediates resistance to cell cycle and DNA damage checkpoint kinase inhibitors.. Front Cell. Dev. Biol..

[106] Trusolino L., Bertotti A. (2012). Compensatory pathways in oncogenic kinase signaling and resistance to targeted therapies: Six degrees of separation.. Cancer Discov.

[107] Senderowicz A.M. (1999). Flavopiridol: The first cyclin-dependent kinase inhibitor in human clinical trials.. Invest New Drugs.

[108] Nemunaitis J.J., Small K.A., Kirschmeier P., Zhang D., Zhu Y., Jou Y.M., Statkevich P., Yao S.L., Bannerji R. (2013). A first-in-human, phase 1, dose-escalation study of dinaciclib, a novel cyclin-dependent kinase inhibitor, administered weekly in subjects with advanced malignancies.. J. Transl. Med..

[109] Seftel M.D., Kuruvilla J., Banerji V., Fraser G., Xu H., Powers J., Crocker S., Toguchi M., Lyons J.F., Seymour L., Hay A.E. (2014). A phase II study of AT7519M, a CDK inhibitor, in patients with chronic lymphocytic leukemia:. NCIC CTG Trial IND. 193. Blood.

[110] House I., Valore-Caplan M., Maris E., Falchook G.S. (2025). Cyclin Dependent Kinase 2 (CDK2) Inhibitors in Oncology Clinical Trials:. A Review. J. Immunother Precis Oncol..

[111] Murphy C.G., Dickler M.N. (2015). The role of CDK4/6 inhibition in breast cancer.. Oncologist.

[112] Deep A., Marwaha R.K., Marwaha M.G., Jyoti J., Nandal R., Sharma A.K. (2018). Flavopiridol as cyclin dependent kinase (CDK) inhibitor: A review.. New J. Chem..

[113] Zhang M., Zhang L., Hei R., Li X., Cai H., Wu X., Zheng Q., Cai C. (2021). CDK inhibitors in cancer therapy, an overview of recent development.. Am. J. Cancer Res..

[114] Zhao W., Zhang L., Zhang Y., Jiang Z., Lu H., Xie Y., Han W., Zhao W., He J., Shi Z., Yang H., Chen J., Chen S., Li Z., Mao J., Zhou L., Gao X., Li W., Tan G., Zhang B., Wang Z. (2023). The CDK inhibitor AT7519 inhibits human glioblastoma cell growth by inducing apoptosis, pyroptosis and cell cycle arrest.. Cell. Death Dis..

[115] Liu Y., Wu J., Ji Z., Chen L., Zou J., Zheng J., Lin W., Cai J., Chen Y., Zheng D., Chen Y., Li Z. (2023). Comparative efficacy and safety of different combinations of three CDK4/6 inhibitors with endocrine therapies in HR+/HER-2 - metastatic or advanced breast cancer patients: a network meta-analysis.. BMC Cancer.

[116] Malyukova A., Lahnalampi M., Falqués-Costa T., Pölönen P., Sipola M., Mehtonen J., Teppo S., Akopyan K., Viiliainen J., Lohi O., Hagström-Andersson A.K., Heinäniemi M., Sangfelt O. (2024). Sequential drug treatment targeting cell cycle and cell fate regulatory programs blocks non-genetic cancer evolution in acute lymphoblastic leukemia.. Genome Biol..

[117] Cai Q., Guo R., Chen D., Deng Z., Gao J. (2025). SynBioNanoDesign: Pioneering targeted drug delivery with engineered nanomaterials.. J. Nanobiotechnology.

[118] Amin S., Bathe O.F. (2016). Response biomarkers: Re-envisioning the approach to tailoring drug therapy for cancer.. BMC Cancer.

[119] Feng X., Li Z., Liu Y., Chen D., Zhou Z. (2024). CRISPR/Cas9 technology for advancements in cancer immunotherapy: From uncovering regulatory mechanisms to therapeutic applications.. Exp. Hematol. Oncol..

[120] Di Carlo E., Sorrentino C. (2024). State of the art CRISPR-based strategies for cancer diagnostics and treatment.. Biomark Res..

[121] Jiménez-Luna J., Grisoni F., Weskamp N., Schneider G. (2021). Artificial intelligence in drug discovery: Recent advances and future perspectives.. Expert Opin. Drug Discov..

[122] Miranda F., Prazeres H., Mendes F., Martins D., Schmitt F. (2022). Resistance to endocrine therapy in HR + and/or HER2+ breast cancer: The most promising predictive biomarkers.. Mol. Biol. Rep.

[123] Sharma V., Singh A., Chauhan S., Sharma P.K., Chaudhary S., Sharma A., Porwal O., Fuloria N.K. (2024). Role of artificial intelligence in drug discovery and target identification in cancer.. Curr. Drug Deliv.

[124] Ozaybi M.Q.B., Madkhali A.N.M. (2024). Alhazmi, M.A.M.; faqihi, H.; Alanazi, M.M.; Siraj, W.H.Y.; Zalah, A.H.A.; Khormi, M.M.A.; al salem, A.; Mashragi, T.Q.M.; Alotaibi, A.N.; Naji, A.A.M.; Bagal, R.M.A.; Maswdi, A.M.M.; Marwee, H.A.A. The Role of. Artificial Intelligence in Drug Discovery and Development. Egypt J. Chem..

[125] Asghar A., Chohan T.A., Khurshid U., Saleem H., Mustafa M.W., Khursheed A., Alafnan A., Batul R., Bin Break M.K., Almansour K., Anwar S. (2024). A systematic review on understanding the mechanistic pathways and clinical aspects of natural CDK inhibitors on cancer progression.: Unlocking cellular and biochemical mechanisms.. Chem. Biol. Interact..

[126] Jumper J., Evans R., Pritzel A., Green T., Figurnov M., Ronneberger O., Tunyasuvunakool K., Bates R., Žídek A., Potapenko A., Bridgland A., Meyer C., Kohl S.A.A., Ballard A.J., Cowie A., Romera-Paredes B., Nikolov S., Jain R., Adler J., Back T., Petersen S., Reiman D., Clancy E., Zielinski M., Steinegger M., Pacholska M., Berghammer T., Bodenstein S., Silver D., Vinyals O., Senior A.W., Kavukcuoglu K. (2021). Kohli, P.;. Hassabis, D. Highly accurate protein structure prediction with AlphaFold. Nature.

[127] Ramsundar B., Eastman P., Walters P., Pande V. (2019). Deep learning for the life sciences: Applying deep learning to genomics, microscopy, drug discovery, and more;. O'Reilly Media: Sebastopol, CA,.

[128] Heid E., Greenman K.P., Chung Y., Li S.C., Graff D.E., Vermeire F.H., Wu H., Green W.H., McGill C.J. (2024). Chemprop: A machine learning package for chemical property prediction.. J. Chem. Inf Model.

[129] Du P., Fan R., Zhang N., Wu C., Zhang Y. (2024). Advances in integrated multi-omics analysis for drug-target identification.. Biomolecules.

[130] Ocana A., Pandiella A., Privat C., Bravo I., Luengo-Oroz M., Amir E., Gyorffy B. (2025). Integrating artificial intelligence in drug discovery and early drug development: A transformative approach.. Biomark Res..

[131] Leng D., Zheng L., Wen Y., Zhang Y., Wu L., Wang J., Wang M., Zhang Z., He S., Bo X. (2022). A benchmark study of deep learning-based multi-omics data fusion methods for cancer.. Genome Biol..

[132] Ballard J.L., Wang Z., Li W., Shen L., Long Q. (2024). Deep learning-based approaches for multi-omics data integration and analysis.. BioData Min.

[133] Badawy M., Ramadan N., Hefny H.A. (2023). Healthcare predictive analytics using machine learning and deep learning techniques: A survey.. J. Electr. Syst. Inf. Technol..

[134] Amiri Z., Heidari A., Navimipour N.J., Unal M., Mousavi A. (2023). Adventures in data analysis: A systematic review of Deep Learning techniques for pattern recognition in cyber-physical-social systems.. Multimedia Tools Appl..

[135] Frasca M., La Torre D., Repetto M., De Nicolò V., Pravettoni G., Cutica I. (2024). Artificial intelligence applications to genomic data in cancer research: A review of recent trends and emerging areas.. Discover Analytics.

[136] Qin R.X., Yang Y., Chen J.F., Huang L.J., Xu W., Qin Q.C., Liang X.J., Lai X.Y., Huang X.Y., Xie M.S., Chen L. (2023). Transcriptomic analysis reveals the potential biological mechanism of AIS and lung adenocarcinoma.. Front. Neurol..

[137] Li J., Wang Y., Wang X., Yang Q. (2020). CDK1 and CDC20 overexpression in patients with colorectal cancer are associated with poor prognosis: Evidence from integrated bioinformatics analysis.. World J. Surg. Oncol..

[138] Shen J., Gong X., Ren H., Tang X., Yu H., Tang Y., Chen S., Ji M. (2025). Identification and validation of CDK1 as a promising therapeutic target for Eriocitrin in colorectal cancer: A combined bioinformatics and experimental approach.. BMC Cancer.

[139] Kim D.N., Yin T., Zhang T., Im A.K., Cort J.R., Rozum J.C., Pollock D., Qian W.J., Feng S. (2024). Artificial intelligence transforming post-translational modification research.. Bioengineering.

[140] Ertelt M., Mulligan V.K., Maguire J.B., Lyskov S., Moretti R., Schiffner T., Meiler J., Schoeder C.T. (2024). Combining machine learning with structure-based protein design to predict and engineer post-translational modifications of proteins.. PLOS Comput. Biol..

[141] Ren F., Ding X., Zheng M., Korzinkin M., Cai X., Zhu W., Mantsyzov A., Aliper A., Aladinskiy V., Cao Z., Kong S., Long X., Man Liu B.H., Liu Y., Naumov V., Shneyderman A., Ozerov I.V., Wang J., Pun F.W., Polykovskiy D.A., Sun C., Levitt M., Aspuru-Guzik A., Zhavoronkov A. (2023). AlphaFold accelerates artificial intelligence powered drug discovery: Efficient discovery of a novel CDK20 small molecule inhibitor.. Chem. Sci..

[142] Guo S.B., Meng Y., Lin L., Zhou Z.Z., Li H.L., Tian X.P., Huang W.J. (2024). Artificial intelligence alphafold model for molecular biology and drug discovery: A machine-learning-driven informatics investigation.. Mol. Cancer.

[143] Aderinwale T., Bharadwaj V., Christoffer C., Terashi G., Zhang Z., Jahandideh R., Kagaya Y., Kihara D. (2022). Real-time structure search and structure classification for AlphaFold protein models.. Commun Biol..

[144] Varadi M., Anyango S., Deshpande M., Nair S., Natassia C., Yordanova G., Yuan D., Stroe O., Wood G., Laydon A., Žídek A., Green T., Tunyasuvunakool K., Petersen S., Jumper J., Clancy E., Green R., Vora A., Lutfi M., Figurnov M., Cowie A., Hobbs N., Kohli P., Kleywegt G., Birney E., Hassabis D., Velankar S. (2022). AlphaFold protein structure database: Massively expanding the structural coverage of protein-sequence space with high-accuracy models.. Nucleic Acids Res..

[145] Neef R., Gruneberg U., Kopajtich R., Li X., Nigg E.A., Sillje H., Barr F.A. (2007). Choice of Plk1 docking partners during mitosis and cytokinesis is. controlled by the activation state of Cdk1. Nat. Cell. Biol..

[146] Kaveh S., Mani-Varnosfaderani A., Neiband M.S. (2024). Deriving general structure-activity/selectivity relationship patterns for different subfamilies of cyclin-dependent kinase inhibitors using machine learning methods.. Sci. Rep..

[147] Naithani U., Guleria V. (2024). Integrative computational approaches for discovery and evaluation of lead compound for drug design.. Front Drug Discov..

[148] Teotia V., Jha P., Chopra M. (2024). Discovery of potential inhibitors of CDK1 by integrating pharmacophore-based virtual screening, molecular docking, molecular dynamics simulation studies, and evaluation of their inhibitory activity.. ACS Omega.

[149] Zhou G., Rusnac D.V., Park H., Canzani D., Nguyen H.M., Stewart L., Bush M.F., Nguyen P.T., Wulff H., Yarov-Yarovoy V., Zheng N., DiMaio F. (2024). An artificial intelligence accelerated virtual screening platform for drug discovery.. Nat. Commun.

[150] Beroza P., Crawford J.J., Ganichkin O., Gendelev L., Harris S.F., Klein R., Miu A., Steinbacher S., Klingler F.M., Lemmen C. (2022). Chemical space docking enables large-scale structure-based virtual screening to discover ROCK1 kinase inhibitors.. Nat. Commun.

[151] Muhsen I.N., Hashmi S.K. (2022). Utilizing machine learning in predictive modeling: what's next?. Bone Marrow Transplant..

[152] Alber M., Buganza Tepole A., Cannon W.R., De S., Dura-Bernal S., Garikipati K., Karniadakis G., Lytton W.W., Perdikaris P., Petzold L., Kuhl E. (2019). Integrating machine learning and multiscale modeling-perspectives, challenges, and opportunities in the biological, biomedical, and behavioral sciences.. NPJ Digit. Med..

[153] Sharma A., Lysenko A., Jia S., Boroevich K.A., Tsunoda T. (2024). Advances in AI and machine learning for predictive medicine.. J. Hum. Genet..

[154] Wells C.I., Vasta J.D., Corona C.R., Wilkinson J., Zimprich C.A., Ingold M.R., Pickett J.E., Drewry D.H., Pugh K.M., Schwinn M.K., Hwang B., Zegzouti H., Huber K.V.M., Cong M., Meisenheimer P.L., Willson T.M., Robers M.B. (2020). Quantifying CDK inhibitor selectivity in live cells.. Nat. Commun.

[155] Liu Y., Grimm M., Dai W., Hou M., Xiao Z.X., Cao Y. (2020). CB-Dock: A web server for cavity detection-guided protein-ligand blind docking.. Acta. Pharmacol. Sin..

[156] Chatterjee A., Walters R., Shafi Z., Ahmed O.S., Sebek M., Gysi D., Yu R., Eliassi-Rad T., Barabási A.L., Menichetti G. (2023). Improving the generalizability of protein-ligand binding predictions with AI-Bind.. Nat. Commun.

[157] Pecina A., Fanfrlík J., Lepšík M., Řezáč J. (2024). SQM2.20: Semiempirical quantum-mechanical scoring function yields DFT-quality protein-ligand binding affinity predictions in minutes.. Nat. Commun..

[158] Wójcikowski M., Ballester P.J., Siedlecki P. (2017). Performance of machine-learning scoring functions in structure-based virtual screening.. Sci. Rep.

[159] Mohamed Abdul Cader J., Newton M.A.H., Rahman J., Mohamed Abdul Cader A.J., Sattar A. (2024). Ensembling methods for protein-ligand binding affinity prediction.. Sci. Rep..

[160] Valous N.A., Popp F., Zörnig I., Jäger D., Charoentong P. (2024). Graph machine learning for integrated multi-omics analysis.. Br. J. Cancer.

[161] Chen S., Zhang O., Jiang C., Zhao H., Zhang X., Chen M., Liu Y., Su Q., Wu Z., Wang X., Qu W., Ye Y., Chai X., Wang N., Wang T., An Y., Wu G., Yang Q., Chen J., Xie W., Lin H., Li D. (2025). Hsieh, C-Y.; Huang, Y.; Kang, Y.; Hou, T.; Pan, P. Deep lead optimization enveloped in protein pocket and its application in designing potent and selective ligands targeting LTK protein.. Nat. Mach. Intell..

[162] Huang L., Xu T., Yu Y., Zhao P., Chen X., Han J., Xie Z., Li H., Zhong W., Wong K.C., Zhang H. (2024). A dual diffusion model enables 3D molecule generation and lead optimization based on target pockets.. Nat. Commun..

[163] Atz K., Cotos L., Isert C., Håkansson M., Focht D., Hilleke M., Nippa D.F., Iff M., Ledergerber J., Schiebroek C.C.G., Romeo V., Hiss J.A., Merk D., Schneider P., Kuhn B., Grether U., Schneider G. (2024). Prospective de novo drug design with deep interactome learning.. Nat. Commun..

[164] Mao X., Chu Y., Wei D. (2024). Designed with interactome-based deep learning.. Nat. Chem. Biol..

[165] Mullard A. (2021). What does AlphaFold mean for drug discovery?. Nat. Rev. Drug Discov..

[166] Yang Z., Zeng X., Zhao Y., Chen R. (2023). AlphaFold2 and its applications in the fields of biology and medicine.. Signal. Transduct Target Ther..

[167] Macedo B., Ribeiro Vaz I., Taveira Gomes T. (2024). MedGAN: Optimized generative adversarial network with graph convolutional networks for novel molecule design.. Sci. Rep..

[168] Du Y., Jamasb A.R., Guo J., Fu T., Harris C., Wang Y., Duan C., Liò P., Schwaller P., Blundell T.L. (2024). Machine learning-aided generative molecular design.. Nat. Mach Intell..

[169] Wu M.H., Xie Z., Zhi D. (2025). A folding-docking-affinity framework for protein-ligand binding affinity prediction.. Commun. Chem..

[170] Liu H., Chen P., Zhai X., Huo K.G., Zhou S., Han L., Fan G. (2024). PPB-Affinity: Protein-Protein binding affinity dataset for ai-based protein drug discovery.. Sci. Data.

[171] Habiballah S., Reisfeld B. (2023). Adapting physiologically-based pharmacokinetic models for machine learning applications.. Sci. Rep..

[172] Hutson M. (2024). How AI is being used to accelerate clinical trials.. Nature.

[173] Zhang B., Zhang L., Chen Q., Jin Z., Liu S., Zhang S. (2023). Harnessing artificial intelligence to improve clinical trial design.. Commun. Med..

[174] Jin Q., Wang Z., Floudas C.S., Chen F., Gong C., Bracken-Clarke D., Xue E., Yang Y., Sun J., Lu Z. (2024). Matching patients to clinical trials with large language models.. Nat. Commun..

[175] Rosenthal J.T., Beecy A., Sabuncu M.R. (2025). Rethinking clinical trials for medical AI with dynamic deployments of adaptive systems.. NPJ Digit. Med..

[176] Yang X., Hu X., Yin J., Li W., Fu Y., Yang B., Fan J., Lu F., Qin T., Kang X., Zhuang X., Li F., Xiao R., Shi T., Song K., Li J., Chen G., Sun C. (2024). Comprehensive multi-omics analysis reveals WEE1 as a synergistic lethal target with hyperthermia through CDK1 super-activation.. Nat. Commun..

[177] Banyai G., Baïdi F., Coudreuse D., Szilagyi Z. (2016). Cdk1 activity acts as a quantitative platform for coordinating cell cycle progression with periodic transcription.. Nat. Commun..

[178] Horejs C.M. (2023). Artificial intelligence identifies new cancer biomarkers.. Nat. Rev. Bioeng..

[179] Overgaard S.M., Graham M.G., Brereton T., Pencina M.J., Halamka J.D., Vidal D.E., Economou-Zavlanos N.J. (2023). Implementing quality management systems to close the AI translation gap and facilitate safe, ethical, and effective health AI solutions.. NPJ Digit. Med..

[180] Hutson M. (2023). Rules to keep AI in check: Nations carve different paths for tech regulation.. Nature.

[181] Zaidan E., Ibrahim I.A. (2024). AI governance in a complex and rapidly changing regulatory landscape: A global perspective.. Humanit Soc. Sci. Commun..

[182] Wang X.Q., Lo C.M., Chen L., Ngan E.S.W., Xu A., Poon R.Y.C. (2017). CDK1-PDK1-PI3K/Akt signaling pathway regulates embryonic and induced pluripotency.. Cell. Death Differ..

[183] Richardson J.P., Smith C., Curtis S., Watson S., Zhu X., Barry B., Sharp R.R. (2021). Patient apprehensions about the use of artificial intelligence in healthcare.. NPJ Digit. Med..

[184] Di Sante G., Pagé J., Jiao X., Nawab O., Cristofanilli M., Skordalakes E., Pestell R.G. (2019). Recent advances with cyclin-dependent kinase inhibitors: therapeutic agents for breast cancer and their role in immuno-oncology.. Expert Rev. Anticancer Ther..

[185] Li J., Huo X., Zhao F., Ren D., Ahmad R., Yuan X., Du F., Zhao J. (2020). Association of cyclin-dependent Kinases 4 and 6 inhibitors with survival in patients with hormone receptor-positive metastatic breast cancer: A systematic review and meta-analysis.. JAMA Netw. Open.

[186] Asciolla J.J., Wu X., Adamopoulos C., Gavathiotis E., Poulikakos P.I. (2025). Resistance mechanisms and therapeutic strategies of CDK4 and CDK6 kinase targeting in cancer.. Nat. Cancer.

[187] Abdelmalak M., Singh R., Anwer M., Ivanchenko P., Randhawa A., Ahmed M., Ashton A.W., Du Y., Jiao X., Pestell R. (2022). The renaissance of CDK inhibitors in breast cancer therapy: An update on clinical trials and therapy resistance.. Cancers.

[188] Wu C.X., Wang X.Q., Chok S.H., Man K., Tsang S.H.Y., Chan A.C.Y., Ma K.W., Xia W., Cheung T.T. (2018). Blocking CDK1/PDK1/β-Catenin signaling by CDK1 inhibitor RO3306 increased the efficacy of sorafenib treatment by targeting cancer stem cells in a preclinical model of hepatocellular carcinoma.. Theranostics.

[189] Di Stefano M., Piazza L., Poles C., Galati S., Granchi C., Giordano A., Campisi L., Macchia M., Poli G., Tuccinardi T. (2025). KinasePred: A computational tool for small-molecule kinase target prediction.. Int. J. Mol. Sci..

[190] Wu J., Chen Y., Wu J., Zhao D., Huang J., Lin M., Wang L. (2024). Large-scale comparison of machine learning methods for profiling prediction of kinase inhibitors.. J. Cheminform.

[191] Yang K., Swanson K., Jin W., Coley C., Eiden P., Gao H., Guzman-Perez A., Hopper T., Kelley B., Mathea M., Palmer A., Settels V., Jaakkola T., Jensen K., Barzilay R. (2019). Analyzing learned molecular representations for property prediction.. J. Chem. Inf. Model.

[192] Bhat A.A., Nisar S., Mukherjee S., Saha N., Yarravarapu N., Lone S.N., Masoodi T., Chauhan R., Maacha S., Bagga P., Dhawan P., Akil A.A.S., El-Rifai W., Uddin S., Reddy R., Singh M., Macha M.A., Haris M. (2022). Integration of CRISPR/Cas9 with artificial intelligence for improved cancer therapeutics.. J. Transl. Med..

